# Fastlim: a fast LHC limit calculator

**DOI:** 10.1140/epjc/s10052-014-3163-1

**Published:** 2014-11-28

**Authors:** Michele Papucci, Kazuki Sakurai, Andreas Weiler, Lisa Zeune

**Affiliations:** 1 Michigan Center for Theoretical Physics, University of Michigan, Ann Arbor, MI 48109 USA; 2 Physics Department, King’s College London, Strand, London, WC2R 2LS UK; 3 CERN TH-PH Division, Meyrin, Switzerland; 4 Deutsches Elektronen-Synchrotron DESY, Notkestraße 85, 22607 Hamburg, Germany

## Abstract

Fastlim is a tool to calculate conservative limits on extensions of the Standard Model from direct LHC searches without performing any Monte Carlo event generation. The program *reconstructs* the visible cross sections (cross sections after event selection cuts) from pre-calculated efficiency tables and cross section tables for simplified event topologies. As a proof of concept of the approach, we have implemented searches relevant for supersymmetric models with R-parity conservation. Fastlim takes the spectrum and coupling information of a given model point and provides, for each signal region of the implemented analyses, the visible cross sections normalised to the corresponding upper limit, reported by the experiments, as well as the $\mathrm{CL}_\mathrm{s}$ value. To demonstrate the utility of the program we study the sensitivity of the recent ATLAS missing energy searches to the parameter space of natural SUSY models. The program structure allows the straightforward inclusion of external efficiency tables and can be generalised to R-parity violating scenarios and non-SUSY models. This paper serves as a self-contained user guide and indicates the conventions and approximations used.

## Introduction

### Motivation

In the 3 years of LHC operation, ATLAS and CMS have conducted many direct new physics searches. These searches have put significant constraints on the parameter space of new physics models. The experimental collaborations have so far interpreted their results in simplified scenarios of full models like the Constrained MSSM (CMSSM) or various simplified models, which are defined by effective Lagrangians with a small number of new physics particles and couplings; see e.g. [1–4]. On the other hand, many models have not been covered and most of the parameter space of the studied models (e.g. the MSSM with ${\sim }20$ phenomenological parameters) has been left unexplored, except for a few very computationally intensive efforts in the MSSM [5–12].

An important question is how sensitive current analyses are to models that have so far been ignored by ATLAS and CMS and if there are holes in the coverage in the models that have been studied. Existing experimental analyses are often sensitive to alternate models, so there is not necessarily any additional effort required for the experiments in the limit setting process – it is only a matter of reinterpreting existing results. While the experimental collaborations can do this, it is often not a good use of their computing resources and the effort required in reinterpreting results could be spent in performing new analyses.

Recently, various groups have started to recast direct LHC searches to extract limits on new physics scenarios; see e.g. [13–34]. However, this usually requires a tedious task for which requires a chain of Monte Carlo (MC) simulations is needed: event generation, detector simulation and efficiency estimation – taking often in total a few hours to test a single model point and a large computing cluster for days to perform parameter scans. Tuning the MC simulations and validating the efficiency estimation for each analysis can also be cumbersome, especially when several analyses are considered.

On the other hand, for models like the MSSM, the idea of Simplified Models provides the basis to decouple the (slow) MC event generation and simulation steps necessary to estimate the efficiencies, from the (much faster) limit setting steps. It is therefore desirable to develop a tool which is simple in use and can calculate a conservative limit in less than a minute per model point by using this principle. We present such a tool (Fastlim) in this paper. We have developed the first version of Fastlim specialising on R-parity conserving supersymmetric models but the approach can be generalised to any new physics model.

A novel feature of the program is that it does not perform any MC simulation to calculate visible cross sections. Instead, the program *reconstructs* the visible cross sections from the contributions of the relevant simplified event topologies. The visible cross section for each event topology^1^ and signal region^2^ is obtained by interpolating the pre-calculated efficiency tables and the cross section tables, which are provided together with the program. In this approach, the reconstructed visible cross section may only be underestimated because only the available simplified topologies and searches are considered. In other words, the limits obtained by Fastlim are always conservative. Including additional topologies may strengthen the bounds.^3^ The first version of Fastlim contains a set of event topologies which can cover the natural SUSY model parameter space. The input of the program are the masses and decay branching ratios of SUSY particles which must be given in the Supersymmetry Les Houches Accord (SLHA) [35, 36] format. The running time is between a couple of seconds and about a half minute depending on the model point and the CPU speed. For a short guide of the installation and a quick start of Fastlim, see Appendices  and .

The paper is organised as follows: the next section describes the method and the calculation procedure of the program. In Sect. 3, the definition of the event topologies and our nomenclature for their identification are given. Section 4 explains the output files, in which the users can find the constraints set by the direct SUSY searches on the input model. Several useful approximations are introduced in Sect. 6, which can be used to enhance the performance of the program when there is a mass degeneracy in the spectrum. Section 7 provides the detailed information on version 1.0. In Sect. 8, we study the direct SUSY search constraints on the natural SUSY models using Fastlim 1.0. Section 9 is dedicated to a summary and future developments.

## Methodology

### The traditional “recasting” approach

In a cut-and-count based analysis, experimentalists define several sets of selection cuts, called *signal regions*, where the SM events are suppressed whilst the signal events are enhanced. One can test any SUSY model by confronting the predicted events by the theory (the sum of the SM and SUSY contributions) with the observed data in the signal regions. The SUSY contribution to the signal region $a$, $N_\mathrm{SUSY}^{(a)}$, can be written as

1 $$\begin{aligned} N_{\mathrm{SUSY}}^{(a)} = \epsilon ^{(a)} \cdot \sigma _\mathrm{SUSY} \cdot \mathcal{L}_\mathrm{int}, \end{aligned}$$

where $\epsilon ^{(a)}$ is the efficiency for the signal region $a$, $\sigma _\mathrm{SUSY}$ is the inclusive SUSY cross section and $\mathcal{L}_\mathrm{int}$ is the integrated luminosity used in the analysis. The efficiency and the cross section depend in general on the whole sparticle mass spectrum and couplings. The SUSY cross section is calculable based on the factorisation theorem and the Feynman diagram approach. Several public tools are available to calculate the total cross section beyond leading order [37–45]. One estimates the efficiency with a MC simulation, according to

2 $$\begin{aligned} \epsilon ^{(a)} = \lim _{N_\mathrm{MC} \rightarrow \infty } \frac{~ \#\mathrm{~of~events~falling~in~signal~region~}a ~}{~ \#\mathrm{~of~generated~events}}. \end{aligned}$$

There are several stages in this calculation. First, SUSY events should be generated using event generators (e.g. Herwig [46–49], Pythia [50, 51] and MadGraph [52]). The event sample is then passed to fast detector simulation codes (e.g. Delphes [53] and PGS [54]) which should be tuned beforehand to correctly reproduce the detector response and object reconstruction criteria for a given analysis. Finally signal region cuts must be implemented, and the efficiency is then estimated according to Eq. (2) using the detector level events.

This method is generic and applicable to any model. However, one has to tune the detector simulation and define the reconstructed objects (often on a per analysis basis), mock-up the analyses and validate the codes in some way. This task becomes increasingly difficult as the analyses become more elaborate and their number and the number of signal regions increases. One of the solutions to this problem would be to develop a program that automatically evaluates efficiencies taking detector effects into account, in which well validated analyses are already implemented together with the appropriate detector setups. Along these lines, ATOM [55] has been developed and already applied to some studies [56, 57].^4^ ATOM also plays a crucial role in developing Fastlim version 1.0 as we will see in Sect. 7. Another issue is the computation time. Even if the efficiencies were automatically calculated, the whole process, including event generation and efficiency evaluation, can easily take tens of minutes to an hour per model point. This becomes a crucial problem when a parameter scan is performed, requiring large computing facilities. To overcome this problem, leveraging on the idea of simplified topologies, we take a different approach, which is described in the next subsection.

### The method

We start by rewriting $N_\mathrm{SUSY}^{(a)}$. The SUSY contribution can be expressed as the sum of the contributions of all event topologies,

3 $$\begin{aligned} N_\mathrm{SUSY}^{(a)} = \sum _i^\mathrm{all~topologies} \epsilon ^{(a)}_i \cdot \sigma _i \cdot \mathcal{L}_\mathrm{int}, \end{aligned}$$

where $\epsilon _i^{(a)}$ is the efficiency for topology $i$, which can be calculated in the same way as in Eq. (2) but using the events with topology $i$ exclusively. Here, we have ignored the interference among the topologies that give the same final states. This approximation is usually very good in weakly coupled BSM theories since the width of BSM particles is generally small and different topologies have different on-shell conditions associated with the intermediate BSM particles. The definition of the event topologies will be illustrated in the example below and is further clarified in Sect. 3. The cross section for topology $i$, $\sigma _i$, can be written by the product of the production cross section and the branching ratios for the decay chains. The visible cross section, $\sigma _\mathrm{vis}^{(a)} \equiv N_\mathrm{SUSY}^{(a)}/\mathcal{L}_\mathrm{int}$, can be written as, for instance,

4 $$\begin{aligned}&\sigma _\mathrm{vis}^{(a)}\nonumber \\&\quad =\epsilon ^{(a)}_{{\tilde{g}}\rightarrow q q {\tilde{\chi }_{1}^{0}}:{\tilde{g}}\rightarrow q q {\tilde{\chi }_{1}^{0}}}(m_{{\tilde{g}}}, m_{{\tilde{\chi }_{1}^{0}}})\cdot \sigma _{{\tilde{g}}{\tilde{g}}}(m_{\tilde{g}}, m_{\tilde{q}}) \cdot (BR_{{\tilde{g}}\rightarrow qq {\tilde{\chi }_{1}^{0}}})^2\nonumber \\&\qquad +\,\epsilon ^{(a)}_{{\tilde{q}}\rightarrow q {\tilde{\chi }_{1}^{0}}: {\tilde{q}}\rightarrow q {\tilde{\chi }_{1}^{0}}}(m_{{\tilde{q}}}, m_{{\tilde{\chi }_{1}^{0}}})\cdot \sigma _{{\tilde{q}}{\tilde{q}}}(m_{\tilde{g}}, m_{\tilde{q}}) \cdot (BR_{{\tilde{q}}\rightarrow q {\tilde{\chi }_{1}^{0}}})^2\nonumber \\&\qquad +\,\epsilon ^{(a)}_{{\tilde{g}}\rightarrow q q {\tilde{\chi }_{1}^{0}}: {\tilde{q}}\rightarrow q {\tilde{\chi }_{1}^{0}}}(m_{\tilde{g}}, m_{{\tilde{q}}}, m_{{\tilde{\chi }_{1}^{0}}})\cdot \sigma _{{\tilde{g}}{\tilde{q}}}(m_{\tilde{g}}, m_{\tilde{q}})\nonumber \\&\qquad \cdot BR_{{\tilde{g}}\rightarrow q q {\tilde{\chi }_{1}^{0}}} \cdot BR_{{\tilde{q}}\rightarrow q {\tilde{\chi }_{1}^{0}}}+\cdots . \end{aligned}$$

Unlike the $\epsilon ^{(a)}$, the $\epsilon _i$ do not depend on all SUSY parameters but only on the masses and couplings of the particles appearing in the topology $i$. Moreover, the dependence of the efficiency on the couplings is usually small [1]. This is because the couplings only modify angular distributions of the final state particles and hardly alter the hardness of the final state objects. Current LHC searches are still inclusive enough to be not too sensitive to these effects. In Eq. (4), the masses relevant to the efficiencies explicitly appear in the brackets.

If the decay chains in the topology $i$ are sufficiency short, the $\epsilon ^{(a)}_i$ may depend only on two or three mass parameters. For such topologies, one can pre-calculate the $\epsilon ^{(a)}_i (\mathbf{m}_i)$ for every grid point in the parameter space, $\mathbf{m}_i = \{m_i^{(1)}, m_i^{(2)},\ldots \}$, and tabulate its values. Once such tables are available, one can obtain the $\epsilon ^{(a)}_i$ by interpolation and then reconstruct the visible cross section according to Eq. (4) without the need of carrying out a MC simulation again. In practice, due to the “curse of dimensionality”, it is computationally feasible to generate the efficiency tables currently only for topologies with two or three different SUSY particles.^5^ Therefore, some of the topologies may be neglected from the formula (4) and in this case the reconstructed visible cross section is underestimated. This means the derived limit is conservative. The detailed information on the currently available efficiency tables is given in Sects. 5 and 7. Additional tables are currently being produced and once available can be downloaded from the Fastlim website (http://cern.ch/fastlim).

Similarly to the pre-calculated $\epsilon _i^{(a)}$, the program contains cross section tables for the various production modes. The cross section is obtained by interpolating the tables during the reconstruction of the visible cross sections. More details of the cross section calculation is given in Sect. 5.

### The calculation procedure

The calculation procedure is as follows:

- The program first goes through all the decay chains starting with the SUSY particles specified in the main program file, fastlim.py, by following the decay modes listed in the input SLHA file. The program collects the branching fraction of each decay mode and calculates the total branching ratios for possible decay chains. In this process, PySLHA [61] is used to extract the masses and branching ratios from the SLHA file.
- The production cross sections are then extracted for a given production mode by interpolating the cross section tables. It then computes the cross sections of the event topologies, $\sigma _i$, by multiplying the production cross sections by the pairs of decay branching ratios. The set of $\sigma _i$ contains interesting information on the model point. The list of the cross sections for the relevant event topologies (sorted from largest to smallest) is therefore given in the output file.
- A loop through all the event topologies is then performed, where the program checks for the presence of the efficiency tables for the event topology under consideration. If the corresponding efficiency tables are found, the efficiencies for all the signal regions are obtained by interpolating the tables.^6^ The visible cross section for the topology, $\sigma _i^{(a)}$, is then calculated by multiplying the cross section and the efficiency. A sum over all the topologies is performed to compute the total visible cross section, $\sigma _\mathrm{vis}^{(a)}$, for the signal region $a$ (the topologies whose efficiency tables are not available are ignored in this sum). The lists of $\sigma _\mathrm{vis}^{(a)}$ and $\sigma _i^{(a)}$ can also be found in the output file.
- Finally the information as regards the signal region $a$ necessary to set a limit is retrieved. Such information has been previously extracted from the experimental papers and it includes the 95 % CL upper limit on the visible cross section (reported by the experimental collaborations using the full likelihood), $\sigma _\mathrm{UL}^{(a)}$, the contribution of the SM background, $N_\mathrm{BG}^{(a)}$, together with its uncertainty, the observed data, $N_\mathrm{obs}^{(a)}$, and the luminosity used for the analysis. A convenient measure for the exclusion is the ratio between the visible cross section and its 95 % CL upper limit
  $$\begin{aligned} R^{(a)} \equiv \frac{\sigma _\mathrm{vis}^{(a)}}{\sigma ^{(a)}_\mathrm{UL}}. \end{aligned}$$
  The model point is excluded at the 95 % CL if $R^{(a)} > 1$. The program may also calculate an approximate $CL_s^{(a)}$ variable by comparing $N_\mathrm{obs}^{(a)}$ and $N_\mathrm{BG}^{(a)} + N_\mathrm{SUSY}^{(a)}$ taking their uncertainties into account using an approximated likelihood $\mathcal {L} = \mathrm{poiss}(N_\mathrm{obs}^{(a)} | N_\mathrm{SUSY}^{(a)} + {\bar{b}}) \cdot \mathrm{gauss}(N_\mathrm{BG}^{(a)},\delta N_\mathrm{BG}^{(a)} \,|\, {\bar{b}})$. The $CL_s^{(a)}$ variable provides a conservative exclusion criterion [62] since it corrects for under-fluctuations of the background. A model point is excluded if $CL_s^{(a)} < 0.05$. We do not combine multiple signal regions between different analyses, since it requires detailed knowledge on the correlations of both systematical and statistical uncertainties. The program outputs $R^{(a)}$ for all the signal regions and provides an approximate $CL_s^{(a)}$ if specified. An interface to RooStats [63] is currently in testing and will be included in a future version.

**Fig. 1:**
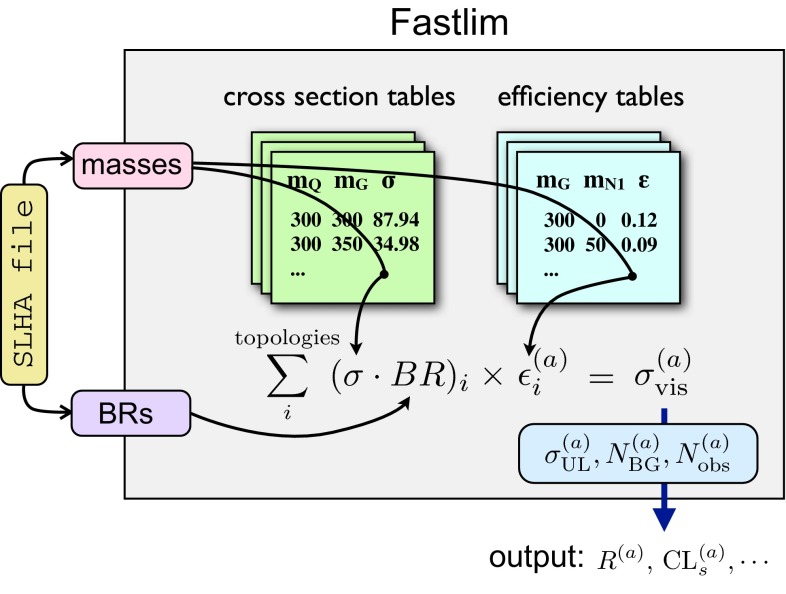
The structure of the program

A schematic diagram for the calculation procedure is shown in Fig. 1.

## Nomenclature of the event topologies

To find an appropriate definition for event topologies and a convenient naming scheme we considered the following points:

- the event topology should be defined such that the efficiency for the topology depends only on the masses of the on-shell SUSY particles appearing in the event topology when the effect of the polarisation and the spin correlation is neglected;
- the definition and classification should be as minimal as possible, otherwise the number of event topologies becomes unreasonably large, requiring unnecessary efficiency tables and slowing down the computation speed;
- the name assigned to the event topology should be as simple and intuitive as possible and must be able to identify the event topology uniquely. It is desirable that the name of event topologies can be directly used as a directory or file name.

Considering the first point in the guideline, the event topology should be defined by not only the final state particles but also the sequences of the intermediate on-shell SUSY particles in the two decay chains. On the other hand, it does not need to specify the interactions and the off-shell particles arising in multi-body or loop-induced decays because they only alter the decay widths and the angular distributions, which do not have a significant impact on the efficiencies in the standard SUSY searches.

We assume that the SUSY particles are pair produced and that each SUSY particle decays into at most one other SUSY particle. This assumption is true for most R-parity conserving models,^7^ but it is also realised in a large class of R-parity violating models, for which the RPV decays are present only at the end of the decay chain, due to the smallness of the RPV couplings. For those models we allow the decay of the lightest SUSY particle (LSP) into SM particles. With this assumption, decay chains can be identified by tracing the decays of SUSY particles from heavier to lighter together with the SM particles produced at each decay. It is therefore convenient to introduce a naming scheme that manifestly distinguishes R-parity even and odd particles. To this end, we use lower case letters for R-even particles and upper case letters for R-odd particles. The names for R-even and R-odd particles are given in Table 1.

**Table 1:** The names for the R-even (top) and R-odd (bottom) particles

Particle	$g$	$\gamma$	$Z$	$h$	$H$	$A$	$W^{\pm }$	$H^{\pm }$	$q$	$t$	$b$	$e$	$\mu$	$\tau$	$\nu$
Name	g	gam	z	h	h2	h3	w	hp	q	t	b	e	m	ta	n

Particle	${\tilde{g}}$	${\tilde{\chi }_{1}^{0}}\cdots {\tilde{\chi }_{4}^{0}}$	${\tilde{\chi }_{1}^{\pm }}, {\tilde{\chi }_{2}^{\pm }}$	${\tilde{q}}$	${\tilde{t}}_1, {\tilde{t}}_2$	${\tilde{b}}_1, {\tilde{b}}_2$	${\tilde{e}}$	${\tilde{\mu }}$	${\tilde{\tau }}_1, {\tilde{\tau }}_2$	${\tilde{\nu }}, {\tilde{\nu }}_{\tau }$					
Name	G	$\mathtt{{N1}} \cdots \mathtt{{N4}}$	C1, C2	Q	T1, T2	B1, B2	E	M	TAU1, TAU2	NU, NUT					

R-parity is not necessarily conserved

**Fig. 2:**
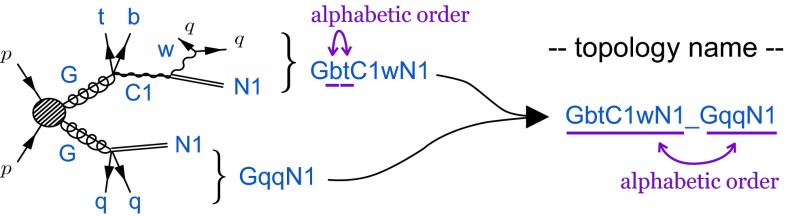
The naming scheme for the event topology

By using the particle names in Table 1, one can assign a unique name to each event topology by connecting the particle names following the two decay chains. Let us consider the event topology $pp \rightarrow {\tilde{g}}{\tilde{g}}$ followed by ${\tilde{g}} \rightarrow qq {\tilde{\chi }_{1}^{0}}$ and ${\tilde{g}}\rightarrow t b {\tilde{\chi }_{1}^{\pm }}, {\tilde{\chi }_{1}^{\pm }}\rightarrow W^{\pm } {\tilde{\chi }_{1}^{0}}$. We give the first decay chain the string $\mathtt{{GqqN1}}$. This string is generated by joining the particle names. In each decay, the mother SUSY particle comes first and daughter SUSY particle comes at the end, if existing. The SM particles are placed right after their mother SUSY particle in alphabetic order. With this rule, the string assigned to the second decay chain is uniquely determined as $\mathtt{{GbtC1wN1}}$. Finally we connect the two strings in the alphabetic order and insert “_” in between, which defines the name $\mathtt{{GbtC1wN1\_GqqN1}}$ for this event topology (see Fig. 2). It is easy to realize that this prescription is unique.

According to our wish list, in order to reduce the length of the decay chains, we do not specify the decay of the SM particles because the decay branching ratios for the SM particles are fixed and independent of the SUSY parameters.^8^ Similarly, we do not specify charges nor do we distinguish particles and anti-particles. This specification is not necessary for our purpose as long as CP is conserved, since the branching ratio is then the same for a process and its CP conjugate. The production cross sections are, on the other hand, different among those processes because the initial $pp$ state at the LHC is not CP invariant. The ratio of the cross sections is, however, fixed once the masses of the produced SUSY particles are given. Consider, for example, $pp \rightarrow {\tilde{d}} {\tilde{u}}^*$ and $pp \rightarrow {\tilde{d}}^* {\tilde{u}}$. The productions are governed by QCD and the cross sections are fully determined by the masses of ${\tilde{u}}$ and ${\tilde{d}}$. The ratio $\sigma ({\tilde{d}} {\tilde{u}}^*)/\sigma ({\tilde{d}}^* {\tilde{u}})$ is therefore fixed if the masses are specified. This means that for each grid point of the efficiency table the ratio between a process and its CP conjugation process is correctly taken into account and is independent of the other parameters. Therefore, the charge of the particle does not need to be specified in the event topology for our purpose. Finally, we also do not yet distinguish between light (s)quark flavours, although the full squark flavour implementation is in principle straightforward. For the effect of large mass splitting between the first two generations, see [57].

Fastlim 1.0 (and the discussion in this section) concentrate on the SUSY models with an (approximate) R-parity symmetry. However, the program is applicable also for non-SUSY models as long as the topology names and the corresponding efficiency tables are provided. Also in that case, the three points in the guideline above provide a useful way to determine the topology names.

## The output

**Fig. 3:**
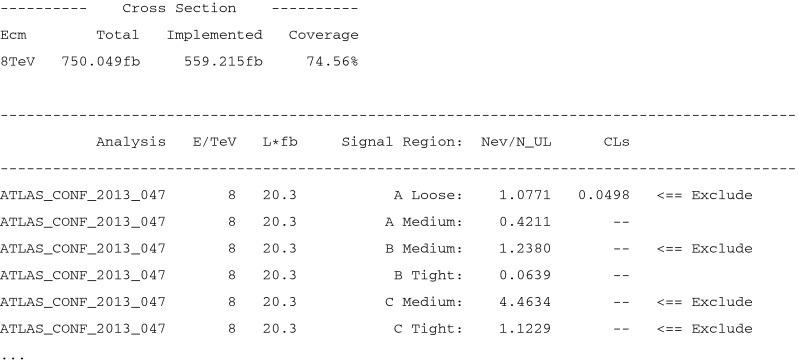
A display output

Users can obtain information on the results at various levels of detail. If the program is executed in the single-model-point input mode (e.g. by ./fastlim.py slha_files/testspectrum.slha), a short summary of the results is displayed on the screen. An example of the display output is shown in Fig. 3. The first piece of information provided is how much of the total cross section is covered by the implemented event topologies. If the cross section of the implemented topologies is substantially smaller than the total SUSY cross section, the limit can become weaker than the “true” limit (the limit with the 100 % coverage). This information is given at the beginning of the display output (see Fig. 3). Below the cross section information, the exclusion measures, $R^{(a)} \equiv N_\mathrm{SUSY}^{(a)}/N_\mathrm{UL}$, are given for all the signal regions. The analysis name, the centre of mass energy, the integrated luminosity $\mathcal{L}_\mathrm{int}$ and the name of signal region are also shown in each line. The $CL_s$ value is only displayed if $|R^{(a)} - 1 | < 0.1$ in the default setup. If $R^{(a)} > 1$, the signal region $a$ excludes the model point at the 95 % CL. In that case, the tag “$<$ == Exclude” appears in the end of the line of that signal region.

**Fig. 4:**
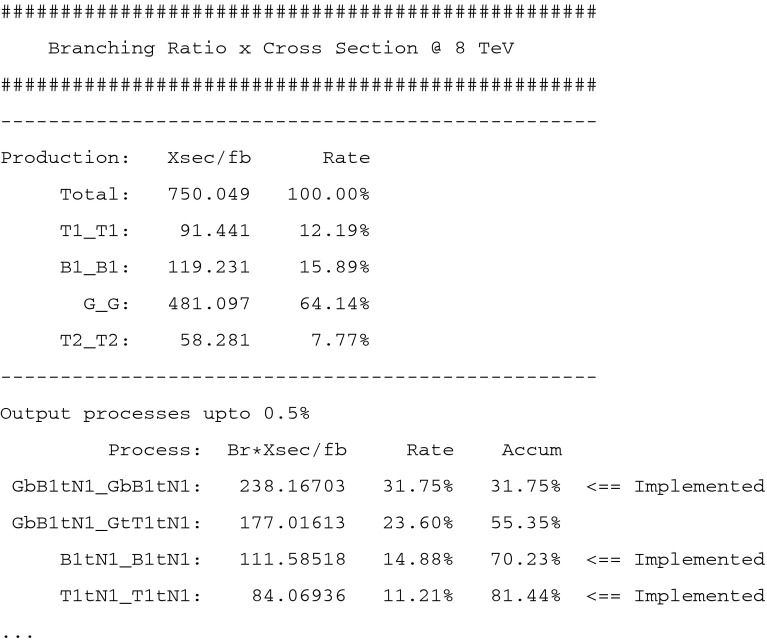
The section dedicated to the cross section times branching ratio in the output file, fastlim.out

For more detailed information, the program also creates the output file, fastlim.out. The first half of an example output file is shown in Fig. 4.

First, the cross section for each production mode is given. Secondly, the list of cross sections (or production cross section times branching ratios) for the relevant event topologies is provided. This list is sorted from the largest cross section to the smallest one. The rate (“Rate”) with which this process contributes to the total cross section and the accumulated rate (“Accum”) up to the topology looked at are also shown. If the efficiency table for a certain event topology is implemented, the tag “$<$ == Implemented” appears.

**Fig. 5:**
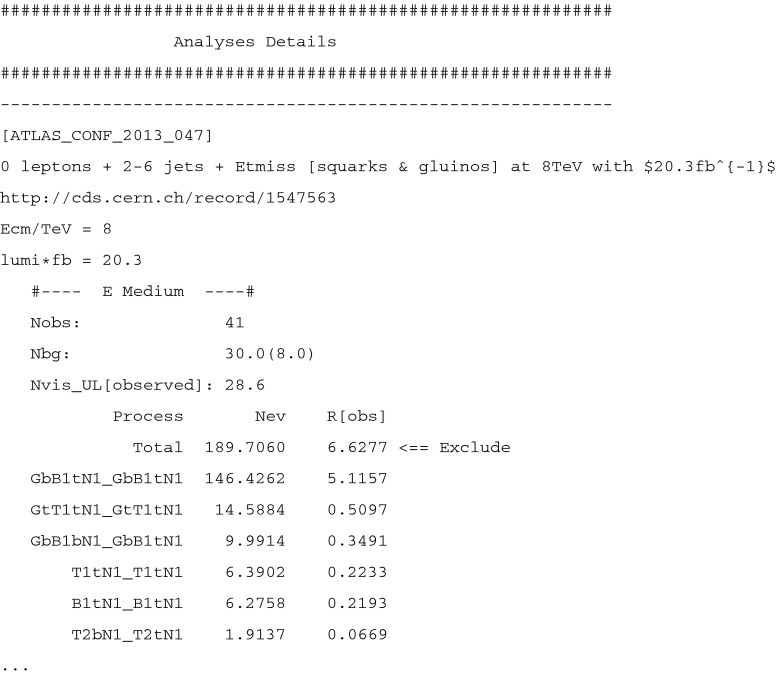
The section dedicated to the information on the analyses and event topology contribution to the signal region in the output file, fastlim.out. E Medium is a label of the signal region

The other half of the output is shown in Fig. 5. In this part the detailed information on the analysis and the constraints can be found. The results, divided into sections, are given for each analysis. Each section starts with the general information, providing a short description of the analysis as well as the web-link to the corresponding paper/note, the centre of mass energy and the integrated luminosity. Subsequently, a summary for each signal region is presented. It provides the name of the signal region, the number of observed events, Nobs, the expected number of SM background events, Nbg, and the 95 % CL upper limit on the SUSY contribution, Nvis_UL $[$ observed $]$. Below this information, the list of contributions of each event topology to the signal region is reported. The event topologies are sorted in descending order from the one with the largest contribution to the smallest one. The contributions to the exclusion measure, R[obs] (=Nev/Nvis_UL[observed]), are also given.

## The numerical tables

The efficiency and cross section tables are provided in the form of a standard text file so that new tables can be added straightforwardly. In this section, we explain the conventions for the efficiency and cross section tables.

### The efficiency tables

**Fig. 6:**
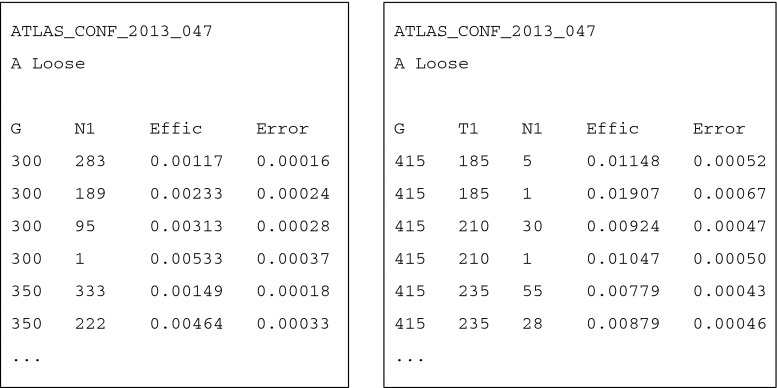
Example efficiency tables for GbbN1_GbbN1/ATLAS_CONF_2013_047/A Loose (*left*) and GtT1tN1_GtT1tN1/ATLAS_CONF_2013_047/A Loose (*right*)

The efficiency table file should be given for each event topology and signal region. Two examples are shown in Fig. 6. The header of the files describes a few remarks about the analysis and the signal region. Below the header, each line provides the efficiency and the MC error for the SUSY masses specified at the beginning of the line from heavier to lighter. The efficiency files are found for instance in
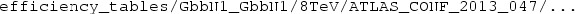

The information as regards the grids can be directly found in the efficiency table files. Although the experimental collaborations have not provided their results of the signal efficiencies for the 2013 SUSY searches, we will include them in our program whenever they will become publicly available. The efficiency tables installed in Fastlim 1.0 are generated by us using MadGraph 5 and ATOM. More detailed information is given in Sect. 7.

### The cross section tables

The cross section tables should be provided for each production mode and the centre of mass energy. In Fastlim 1.0, ${\tilde{g}}{\tilde{g}}$, ${\tilde{g}}{\tilde{q}}$, ${\tilde{q}} {\tilde{q}}$ and ${\tilde{q}} {\tilde{q}}^*$ cross sections and uncertainties are generated by NLL fast [38] combining different PDF sets, following the prescription described in Ref. [64]. For the stop and sbottom pair productions, the cross sections are taken from the values given by the SUSY Cross Section Working Group [65]. The cross section table files are found for example in 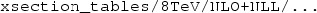


or 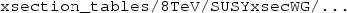


## The approximations

### Treatment of soft decays

Several SUSY models predict partially degenerate SUSY mass spectra. For example, in anomaly mediation, the wino often becomes the lightest SUSY state. Since the wino is SU(2) triplet, it leads to almost degenerate ${\tilde{\chi }_{1}^{\pm }}$ and ${\tilde{\chi }_{1}^{0}}$. Another example is the higgsino LSP scenario. In this case, two higgsino doublets have similar masses, leading to almost degenerate ${\tilde{\chi }_{1}^{\pm }}$, ${\tilde{\chi }_{2}^{0}}$ and ${\tilde{\chi }_{1}^{0}}$.

If one SUSY particle decays to another which has a similar mass, the SM particles produced in the decay will tend to be very soft. Such SM particles may not be observed in the detector because of the low detector acceptance and the reconstruction efficiencies. Even if such objects are reconstructed, they hardly affect the signal region efficiency because the high-$p_T$ cuts employed in the SUSY searches are likely to ignore such objects. Therefore, barring the case of dedicated analyses looking for such soft objects or having low $p_T$ jet vetos, if there is an event topology containing a decay associated with two nearly degenerate SUSY particles, it may be useful to truncate the decay from the topology and redefine it as a shorter effective event topology.

Let us consider e.g. the topology GbbC1qqN1_GbbC1q qN1. If the chargino, C1, and the neutralino, N1, are mass degenerate, its efficiencies would be very similar to those for GbbN1_GbbN1 because the light quarks from the chargino decays will be too soft to be separated from soft QCD radiation. This observation is important because even if the efficiency tables for GbbC1qqN1_GbbC1qqN1 are not available, one can nevertheless extract the efficiency from the GbbN1_GbbN1 efficiency table, if it is implemented. To allow this approximation, we have implemented a Replace() function. In the example above the function can be used as 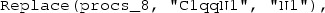


where procs_8 contains the information of all the relevant topologies together with their 8 TeV cross sections (as a Python dictionary). The above command replaces the string C1qqN1 by N1 in all topologies stored in procs_8. If the event topology name generated after this truncation already exists, the contributing cross sections are summed: for the above example the cross section of GbbC1qqN1_GbbC1qqN1 is added to the cross section of GbbN1_GbbN1 and the topology GbbC1qqN1_GbbC1qq N1 is removed from procs_8. In the current version of the program such possibility is implemented by default for N1, N2 and C1 if their mass splitting is smaller than 10 GeV. The extension of such checks to other cases, via a user-defined input file is planned for the next release of Fastlim.

Note that this replacement may introduce topologies in which the electric charge appears not to be conserved.^9^ For example, truncating C1qqN1 in GbbN1_GbtC1qqN1 introduces GbbN1_GbtN1. As will be discussed in Sect. 7.3, the program contains many such event topologies to increase the applicability to concrete models.

### Topologies with similar decay structure

There are several event topologies among which the same efficiency table can be used. An obvious example is T1tN1_ T1tN1 and T2tN1_T2tN1. In general $\tilde{t}_{2}$ and $\tilde{t}_{1}$ decay kinematics depend on their $\tilde{t}_{L,R}$ admixture. The top quarks coming from stop decays may be polarised depending on the $\tilde{t}_{L,R}$ admixture of the stop. This is also known to affect the efficiencies of certain analyses to some level [66, 67]. While including top polarisation is a straightforward addition to Fastlim code (which will be included in later versions), at the moment we provide efficiencies for unpolarized tops only. This allows us to present an example of another simplification feature of the Fastlim code.

Because the polarisation effect is ignored in our calculation, the efficiencies of the two topologies are identical apart from the stop mass. As will be discussed in Sect. 7.3, we provide the efficiency tables only for T1tN1_T1tN1 but use them both for T1tN1_T1tN1 and T2tN1_T2tN1. The same efficiency tables can also be used for B1tN1_B1tN1 and B2tN1_B2tN1, which may arise after truncating the soft chargino decays in B1tC1qqN1_B1tC1qqN1 and B2tC1qqN1_B2tC1qqN1, respectively.

### Reduction of multidimensional topologies

Let us finally consider the case of GtT1tN1_GtT2tN1. This event topology involves four on-shell SUSY particles: G, T2, T1, N1, and in principle requires four-dimensional efficiency tables. However, if e.g. the masses of T1 and T2 are close to each other, one may use the efficiency tables for GtT1tN1_GtT1tN1, which are three dimensional. By default, the efficiencies for GtT1tN1_GtT2tN1 are taken from those for GtT1tN1_GtT1tN1 if $(m_\mathtt{{T2}} - m_\mathtt{{T1}})/m_\mathrm{T2} < 0.1$. The average mass, $(m_\mathtt{{T2}} + m_\mathtt{{T1}})/2$, is used for the mass of the intermediate particle between G and N1 in the interpolation. This approximation can be performed automatically for particles sharing the same type of decay modes. The same procedure and condition are used for instance for GbB1bN1_GbB2bN1 and GbB1bN1_GbB1bN1. As in the case of soft decays, we plan to provide additional user control over this feature in the next Fastlim version by suitable input configuration files.

## Fastlim version 1.0

### Generation of efficiency tables

The simplified model efficiency tables for the 2013 SUSY searches have yet to be provided by the experimental collaborations. The tables included in Fastlim 1.0 have therefore been pre-calculated by us using ATOM. The calculation procedure we used is as follows: $5 \cdot 10^4$ events are generated using MadGraph 5.12 [52] for each grid point in the respective SUSY mass plane (independent of the topology and the mass spectrum). The samples include up to one extra hard parton emission at the matrix element level, matched to the parton shower (carried out by Pythia 6.426 [50]) using the MLM merging scheme [68], where the merging scale is set to $m_\mathrm{SUSY}/4$ with $m_\mathrm{SUSY}$ being the mass of the heavier SUSY particles in the production.

The event files are then passed to ATOM [55], which evaluates the efficiencies for various signal regions taking detector effects into account. ATOM estimates the efficiencies for many implemented signal regions. We have validated the implementation of the analyses in ATOM using the cut-flow tables provided by ATLAS. The validation results are given in Appendix  and the Fastlim website (http://cern.ch/fastlim).

### The available analyses

Most of the standard MET-based searches conducted by ATLAS in 2013 are available in Fastlim version 1.0. The list of the available analyses together with short descriptions, the centre of mass energies, the luminosities and the number of signal regions in the analysis are listed in Table 2. The SUSY searches conducted by CMS will be included in a future update.

**Table 2:** The analyses available in Fastlim version 1.0

Name	Short description	$E_\mathrm{CM}$	$\mathcal{L}_\mathrm{int}$	# SRs	Refs.
ATLAS_CONF_2013_024	0 lepton $+$ 6 (2 b-)jets $+$ MET [Heavy stop]	8	20.5	3	[69]
ATLAS_CONF_2013_035	3 leptons $+$ MET [EW production]	8	20.7	6	[70]
ATLAS_CONF_2013_037	1 lepton $+$ 4(1 b-)jets $+$ MET [Medium/heavy stop]	8	20.7	5	[71]
ATLAS_CONF_2013_047	0 leptons $+$ 2–6 jets $+$ MET [squarks & gluinos]	8	20.3	10	[72]
ATLAS_CONF_2013_048	2 leptons ($+$jets) $+$ MET [Medium stop]	8	20.3	4	[73]
ATLAS_CONF_2013_049	2 leptons $+$ MET [EW production]	8	20.3	9	[74]
ATLAS_CONF_2013_053	0 leptons $+$ 2 b-jets $+$ MET [Sbottom/stop]	8	20.1	6	[75]
ATLAS_CONF_2013_054	0 leptons $+$ $\ge$7–10 jets $+$ MET [squarks and gluinos]	8	20.3	19	[76]
ATLAS_CONF_2013_061	0–1 leptons $+$ $\ge$3 b-jets $+$ MET [3rd gen. squarks]	8	20.1	9	[77]
ATLAS_CONF_2013_062	1–2 leptons $+$ 3–6 jets $+$ MET [squarks and gluinos]	8	20.3	13	[78]
ATLAS_CONF_2013_093	1 lepton $+$ bb(H) $+$ Etmiss [EW production]	8	20.3	2	[79]

The units for the centre of mass energy, $E_\mathrm{CM}$, and the integrated luminosity, $\mathcal{L}_\mathrm{int}$, are TeV and fb$^{-1}$, respectively. The number of signal regions in each analysis and the references are also shown

### The implemented event topologies

**Fig. 7:**
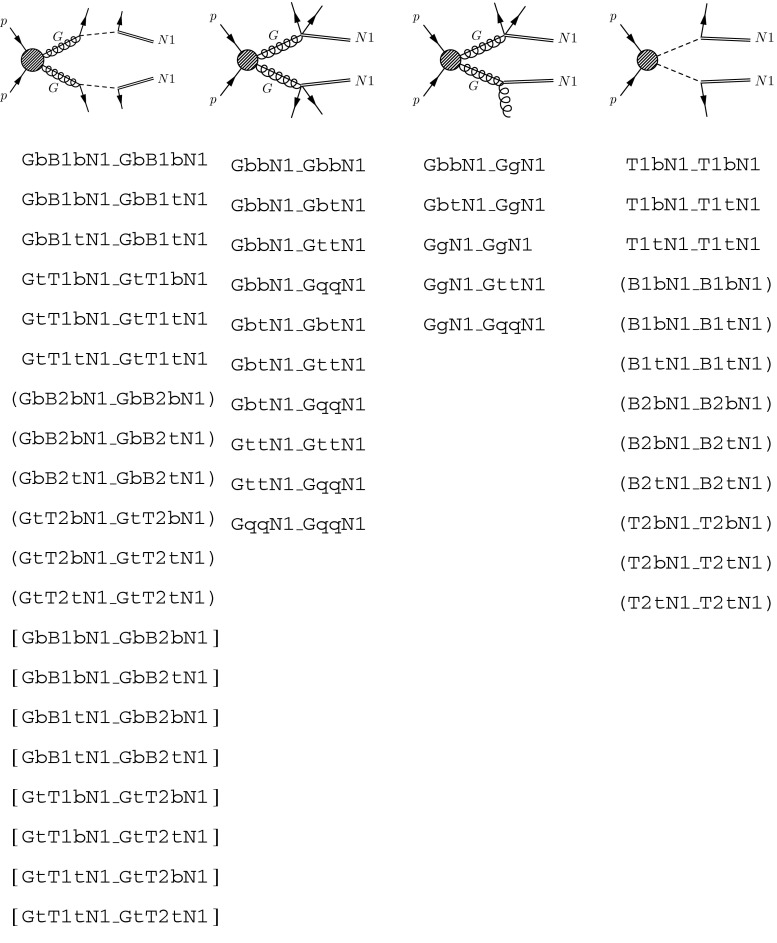
The event topologies whose efficiency tables are implemented in Fastlim version 1.0. The *parentheses* mean that the efficiencies for the topology can be taken from the efficiency tables for one of the other topologies in the same group. On the other hand, the *square bracket* means that the efficiencies can be obtained only when the two intermediate SUSY masses are close $m_{\mathtt{{B1}}} \simeq m_{\mathtt{{B2}}}$ or $m_{\mathtt{{T1}}} \simeq m_{\mathtt{{T2}}}$ (see Sect. 6.2 for more details)

Fastlim 1.0 contains the efficiency tables for a set of event topologies that can cover the *natural SUSY model* parameter space. By *natural SUSY models* we mean a type of spectra where only the gluino, left- and right-handed stops, left-handed sbottom and two higgsino doublets (${\tilde{g}}$, ${\tilde{t}}_R$, ${\tilde{t}}_L$, ${\tilde{b}}_L$, ${\tilde{h}}_u$ and ${\tilde{h}}_d$) reside below a TeV scale and the other SUSY particles are decoupled at the LHC energy scale. To be more precise we list the set of event topologies implemented in Fastlim 1.0 in Fig. 7. In Fig. 7, the parentheses mean that the efficiencies for the topology can be taken from one of the other topologies in the same group. On the other hand, the square bracket means that the efficiencies of the event topology can be obtained only when the condition $m_{\mathtt{{B1}}} \simeq m_{\mathtt{{B2}}}$ or $m_{\mathtt{{T1}}} \simeq m_{\mathtt{{T2}}}$ is satisfied (see Sect. 6.2 for more details).

There are several event topologies in which the electric charge appears not to be not conserved. These topologies can arise after the soft decays are truncated as mentioned in Sect. 6.1. We also include the loop induced ${\mathtt{{G}} \rightarrow \mathtt{{gN1}}}$ decay, which can have a sizeable branching fraction if the two-body modes and GttN1 are kinematically forbidden. The decay rate is also enhanced if the stop and higgsino masses are small and the trilinear $A_t$ coupling is large. These conditions can often be found in natural SUSY models.

Although the event topologies are chosen to cover natural SUSY models, many of the topologies appear also in other models. A large rate of the gluino pair production is relatively common in a wide range of the SUSY models because of the largest colour factor of the gluino among the MSSM particles. Many models tend to predict light stops, since the interaction between the Higgs and stops (with a large top Yukawa coupling) pulls the stop mass down at low energies through the renormalisation group evolution, leading to larger branching ratios for GtT1tN1 and GttN1. The set of the event topologies implemented in Fastlim 1.0 has a very good coverage also for split SUSY models if the wino or the bino is heavier than the gluino.

Additional topologies are currently being evaluated and it will be possible to download them from the Fastlim website (http://cern.ch/fastlim) as they will become available. Furthermore, any additional 3rd-party efficiency map for a topology not currently covered by Fastlim can be easily added by formatting a text file according to the criteria exposed in Sect. 5.1. This is particularly useful to incorporate the efficiency maps that will be available from [80].

## The constraint on natural SUSY models

In this section, we study the direct SUSY search constraints on the natural SUSY models using Fastlim. Since this is a well studied region of the SUSY parameter space [33, 56, 81–88]. it provides a good test case to illustrate the usage of the program.

We define natural SUSY models as a class of spectra where only gluino, left- and right-handed stops, left-handed sbottom and higgsinos are at energy scales accessible by the LHC. These particles are especially sensitive to the tuning in the electroweak symmetry breaking condition,

5 $$\begin{aligned} m_Z^2 = -2(m_{H_u}^2+|\mu |^2) + \mathcal{O}(\cot ^2 \beta ). \end{aligned}$$

This condition implies that both the higgsino mass, $\mu$, and the soft mass of the up-type Higgs, $m_{H_u}$, should not be too far from the $m_Z$ scale at the electroweak scale, otherwise a precise cancellation is required among these parameters. The $m_{H_u}$ receives one-loop corrections that are proportional to the soft masses of the right-handed stop, $M_{U_3}$, and the third generation left-handed quark doublet, $M_{Q_3}$. The $m_{H_u}$ also receives a two-loop correction proportional to the gluino mass, $m_{\tilde{g}}$. From the naturalness point of view, we roughly expect $|\mu | \lesssim M_{U_3}, M_{Q_3} \lesssim m_{\tilde{g}}$. The other sparticles are not very sensitive to the fine tuning condition (5). For the study below we fix the other soft masses at 3 TeV. We calculate the sparticle spectrum and branching ratios using SUSY-HIT [89]. For the results in this section, we generated two-dimensional grids (with ${\sim }500$–$1000$ points) covering slices of natural SUSY parameter space. The constraints presented below are obtained by interpolating (with Mathematica) between the grid points. By using Fastlim performing the whole study with 4,836 parameter points took 18.7 h (14 s per model point on average) on a single computer (single core, 2.4 GHz clock speed).

**Fig. 8:**
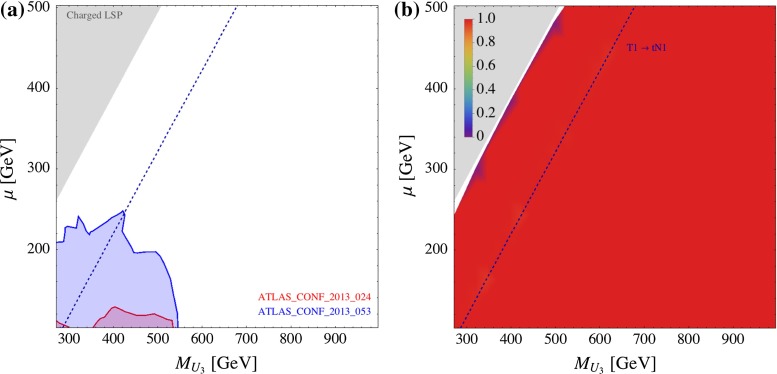
Constraints from direct SUSY searches on the ($M_{U_3}$, $\mu$) plane. The other parameters are $m_{\tilde{g}}=M_{Q_3}=M_{D_3}=3,000$ GeV, $\tan \beta = 10$ and $X_t=0$. The *left plot* shows the exclusion regions from the analyses listed in the plot. The *right plot* shows the cross section coverage, as defined in Eq. (6). The *blue dashed line* represents the kinematical threshold of the ${\mathtt{{T1}} \rightarrow \mathtt{{tN1}}}$ decay

In Fig. 8, we show the direct SUSY search constraints on the ($M_{U_3}$, $\mu$) plane. We fix the other parameters as: $M_{Q_3} = m_{\tilde{g}}= 3$ TeV, $\tan \beta = 10$, $X_t \equiv A_t - \mu \cot \beta = 0$. For one specific analysis the 95 % CL exclusion is obtained by comparing the calculated value for the visible cross section for a certain parameter point with its 95 % CL upper limit in the signal region which has the highest sensitivity. We do not combine several signal regions. In the left plot of Fig. 8 (and in the following plots of that type) we show (superimposed) the 95 % CL exclusion regions from several analyses. Figure 8b shows the cross section coverage

6 $$\begin{aligned} \mathrm{Coverage}=\frac{\sum _{i}^\mathrm{implemented}\sigma _i}{ \sigma _\mathrm{tot}}, \end{aligned}$$

where the numerator is the sum of the cross sections of the topologies implemented in Fastlim 1.0. As can be seen, Fastlim 1.0 has an almost perfect coverage on this parameter slice. In this model, the dominant processes are T1bN1_T1bN1, T1bN1_T1tN1 and T1tN1_T1tN1 after truncating the soft decays among the higgsino states: ${\mathtt{{C1, N2}} \rightarrow \mathtt{{N1}}}$. The three decays are governed by the top Yukawa coupling, but the phase space and symmetry factors give $\sigma ({\mathtt{{T1bN1\_T1tN1}}}) > \sigma ({\mathtt{{T1bN1\_T1bN1}}}) > \sigma ({\mathtt{{T1tN1\_T1tN1}}})$ in most of the parameter region. The blue dashed line represents the kinematical limit of the ${\mathtt{{T1}} \rightarrow \mathtt{{tN1}}}$ decay. The ${\mathtt{{T1bN1\_T1bN1}}}$ dominates in the LHS of this line. In the grey region, the ${\tilde{t}}_1$ becomes lighter than the ${\tilde{\chi }_{1}^{0}}$ and the spectrum has a charged LSP. We therefore do not consider this region.

Figure 8a shows the constraints from all the SUSY searches implemented in Fastlim 1.0 (see Table 2). In this plot (and the following ones of the same type) only the names of the analyses providing an exclusion are listed on the plot, using the same colour as the exclusion contour. The exclusion regions are plotted on top of each other. As can be seen, only ATLAS_CONF_2013_024 and ATLAS_CONF_2013_053 exclude the parameter region in the plot. ATLAS_CONF_2013_024 is designed to constrain the T1tN1_T1tN1 topology focusing on the hadronic top decays. Because T1tN1_T1tN1 is subdominant in this model, the constraint from this analysis is slightly weaker than the corresponding exclusion plot in Ref. [69] assuming $Br( {\tilde{t}}_1 \rightarrow t {\tilde{\chi }_{1}^{0}}) = 1$. ATLAS_CONF_2013_053, on the other hand, has been originally designed for the B1bN1_B1bN1 topology. In this model, T1bN1_T1bN1 has the largest or the second largest rate among the possible topologies depending on the parameter region, and the constraint is quite strong. It roughly excludes $M_{U_3} < 500$ GeV with $\mu < 200$ GeV.

**Fig. 9:**
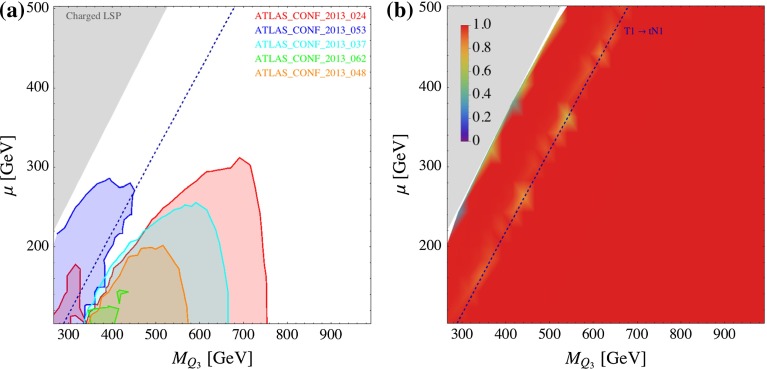
Constraints from direct SUSY searches on the ($M_{Q_3},\mu$) plane. The other parameters are $m_{\tilde{g}}=M_{U_3}=M_{D_3}=3,000$ GeV, $\tan \beta = 10$ and $X_t=0$. The *left plot* shows the exclusion regions from the analyses listed in the plot. The *right plot* shows the cross section coverage, as defined in Eq. (6). The *blue dashed line* represents the kinematical threshold of the ${\mathtt{{T1}} \rightarrow \mathtt{{tN1}}}$ decay

Figure 9 shows the exclusion (left panel (a)) and the cross section coverage (right panel (b)) for the ($M_{Q_3}$, $\mu$) plane. The other parameters are taken as $M_{U_3} = m_{\tilde{g}}= 3$ TeV, $X_t = 0$ and $\tan \beta = 10$. The small $M_{Q_3}$ values result in both light ${\tilde{t}}_L$ and light ${\tilde{b}}_L$. The ${\tilde{t}}_L$ is slightly heavier than the ${\tilde{b}}_L$ because of the contribution from the top quark mass $m_{{\tilde{t}}_L}^2 \simeq M_{Q_3}^2 + m_t^2$. The ${\tilde{t}}_L$ and ${\tilde{b}}_L$ preferably decay to $t_R$ and ${\tilde{h}}_u$ through the interaction term $\mathcal{L} \ni \epsilon ^{\alpha \beta } y_t \bar{t}_R ({\tilde{t}}_L, {\tilde{b}}_L)_{\alpha } ({\tilde{h}}_u^+, {\tilde{h}}_u^0)_{\beta }$. The ${\mathtt{{T1}} \rightarrow \mathtt{{b N1}}}$ and ${\mathtt{{B1}} \rightarrow \mathtt{{b N1}}}$ modes are instead suppressed by the bottom Yukawa coupling. In Fig. 9b, the coverage is slightly off from 100 % near the ${\mathtt{{T1}} \rightarrow \mathtt{{t N1}}}$ kinematical threshold line. In this region, the three-body ${\mathtt{{T1}} \rightarrow \mathtt{{qq B1}}}$ decay via an off-shell $W$ boson takes a small branching fraction. On the left hand side of the blue dashed line, ${\mathtt{{T1bN1\_T1bN1}}}$ and ${\mathtt{{B1bN1\_B1bN1}}}$ dominate.

From Fig. 9a, one can see that ATLAS_CONF_2013_053 only constraints the left hand side of the blue dashed line. This can be understood because the analysis is tailored for the ${\mathtt{{T1bN1\_T1bN1}}}$ and ${\mathtt{{B1bN1\_B1bN1}}}$ topologies. On the other side of the blue dashed line, the ${\mathtt{{T1tN1\_T1tN1}}}$ and ${\mathtt{{B1tN1\_B1tN1}}}$ topologies dominate. In this region, ATLAS_CONF_2013_024 and ATLAS_CONF_2013_037 are particularly constraining because they are designed for the hadronic–hadronic and hadronic–leptonic top modes for the ${\mathtt{{T1tN1\_T1tN1}}}$ topology, respectively. ATLAS_CONF_2013_024 excludes $M_{Q_3}$ values from $\sim$400 up to 750 GeV for $\mu \lesssim 250$ GeV at the $95~\%$ CL. Because of the transition between different dominant decay modes, there is a gap in the exclusion region near the blue dashed line. In this particular region, $M_{Q_3} = 400$ GeV and $\mu = 200$ GeV is still allowed by all the analyses implemented in Fastlim.

**Fig. 10:**
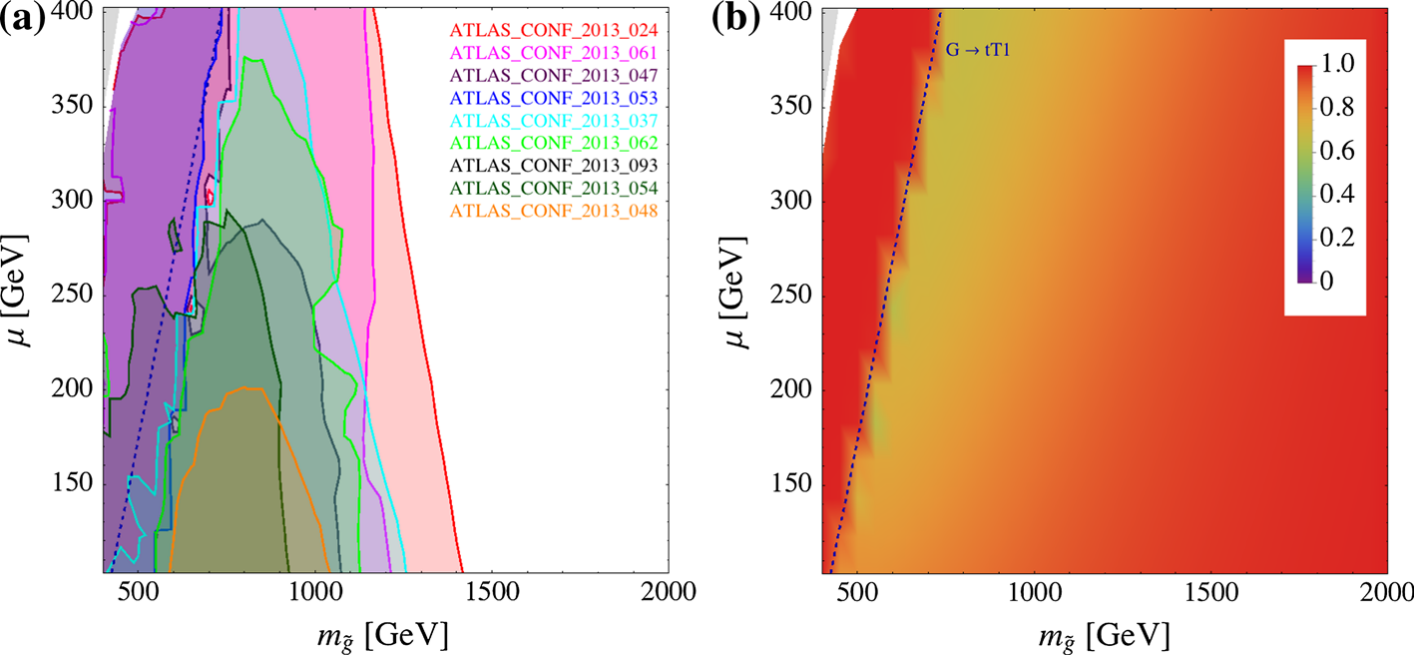
Constraints from direct SUSY searches on the ($m_{\tilde{g}},\mu$) plane. The other parameters are $M_{U_3}=M_{D_3}=3{,}000$ GeV, $\tan \beta = 10$ and $X_t=0$. $M_{Q_3}$ is chosen such that the ${\tilde{t}_1}$ mass is in the middle between the $\tilde{g}$ and $\tilde{\chi }^0_1$ mass ($M_{Q_3}\simeq (m^2_{{\tilde{t}}_1}-m_t^2)^{1/2}$ with $m_{{\tilde{t}}_1} = (m_{\tilde{g}}+ \mu )/2$). The *left plot* shows the exclusion regions from the analyses listed in the plot. The *right plot* shows the cross section coverage, as defined in Eq. (6). The *blue dashed line* represents the kinematical threshold of the ${\mathtt{{G}} \rightarrow \mathtt{{tT1}}}$ decay

Figure 10 shows the exclusion (left panel (a)) and the cross section coverage (right panel (b)) in the ($m_{\tilde{g}}$, $\mu$) plane. Here, we take $M_{U_3} = 3$ TeV, $\tan \beta = 10$, $X_t = 0$. $M_{Q_3}$ is chosen such that the ${\tilde{t}}_1$ mass is in the middle between the ${\tilde{g}}$ and ${\tilde{\chi }_{1}^{0}}$ mass: $M_{Q_3} \simeq (m^2_{{\tilde{t}}_1} - m^2_t )^{1/2}$ with $m_{{\tilde{t}}_1} = (m_{\tilde{g}}+ \mu )/2$. This condition links the stop and sbottom masses to the gluino and higgsino masses, as can be seen from the kinematical threshold for the ${\mathtt{{G}} \rightarrow \mathtt{{tT1}}}$ decay and the charged LSP region which appears in the up left region. Figure 10a shows that the coverage degrades to 70 % near the ${\mathtt{{G}} \rightarrow \mathtt{{t T1}}}$ threshold line, on its right hand side. In this region, asymmetric gluino decays e.g. ${\mathtt{{GbB1tN1\_GtT1tN1}}}$ are relevant, but they are not implemented in Fastlim 1.0 since they require four-dimensional grids.

Nevertheless, one can see from Fig. 10a that many analyses provide exclusion regions in this parameter slice because of the large cross section of the gluino pair production. Among them, ATLAS_CONF_2013_024 and ATLAS_CONF_2013_061 yield the most stringent constraints. ATLAS_CONF_2013_024 mainly constrains T1tN1_T1tN1 and B1tN1_B1tN1 topologies, and the bound on the gluino mass gradually decreases as the stop and sbottom masses increase together with the higgsino mass. On the other hand, the limit from ATLAS_CONF_2013_061 is almost independent of the higgsino mass. This analysis looks for the events with 0–1 lepton plus ${\ge }3$ $b$-jet, targeting the gluino pair production processes with gluino decaying to the third generation quarks either through an on- and off-shell ${\tilde{t}}_1$ and ${\tilde{b}}_1$. The analysis roughly excludes 1.2 TeV gluino regardless of the $\mu$ parameter at the $95~\%$ CL.

We now look at the constraint on the ($m_{\tilde{g}}$, $M_{U_3/Q_3}$) plane, where we take $M_{U_3} = M_{Q_3}$, $\mu = 200$ GeV, $\tan \beta = 10$, $X_t = 0$. Figure 11b shows that the cross section coverage can become as small as 60 % at the vicinity of the ${\mathtt{{G}} \rightarrow \mathtt{{t T1}}}$ threshold line. In this region, again, the asymmetric gluino decays (e.g. GbB1bN1_GtT1tN1 in the region slightly above the ${\mathtt{{G}} \rightarrow \mathtt{{t T1}}}$ threshold line, and e.g. GbB1bN1_GttN1 slightly below the line) become sizeable. One can see from Fig. 11a that the exclusions on the gluino mass and the stop mass are roughly independent of each other. The gluino mass is excluded up to 1280 GeV, almost independently of the stop mass.^10^ The most stringent constraint comes from ATLAS_CONF_2013_061. Near the ${\mathtt{{G}} \rightarrow \mathtt{{t T1}}}$ threshold line the exclusion is degraded because Fastlim 1.0 does not include the topologies with asymmetric gluino decays, though the degradation is only ${\sim }100$ GeV on the gluino mass. The soft mass parameters for the third generation squarks are, on the other hand, constrained up to 750 GeV. ATLAS_CONF_2013_024 provides the strongest limit in the region where $m_{\tilde{g}}> 1.2$ TeV, by excluding the stop production processes independently of the gluino mass.

**Fig. 12:**
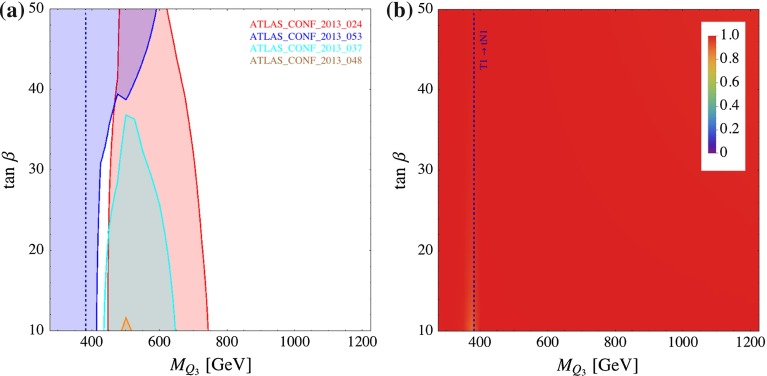
Constraints from direct SUSY searches on the ($M_{Q_3},\tan \beta$) plane. The other parameters are $M_{D_3}=M_{U_3}=m_{\tilde{g}}=3{,}000$ GeV, $\mu =200$ GeV and $X_t=0$. The *left plot* shows the exclusion regions from the analyses listed in the plot. The *right plot* shows the cross section coverage, as defined in Eq. (6). The *blue dashed line* represents the kinematical threshold of the ${\mathtt{{T1}} \rightarrow \mathtt{{tN1}}}$ decay

In Fig. 12, we show the $\tan \beta$ dependence on the $M_{Q_3}$ limit. In this parameter plane, the cross section coverage is ${\sim }100~\%$ across the parameter space. The other parameters are fixed as $\mu = 200$ GeV, $X_t = 0$ and $M_{U_3} = m_{\tilde{g}}= 3$ TeV. This parameter plane intersects that of Fig. 9a at $\mu = 200$ GeV, $\tan \beta = 10$. The gap observed in Fig. 9a around $M_{Q_3} \simeq 400$ GeV, $\mu = 200$ GeV is also seen here. The size of $\tan \beta$ affects the branching fractions of the ${\mathtt{{T1}} \rightarrow \mathtt{{b N1}}}$ and ${\mathtt{{B1}} \rightarrow \mathtt{{b N1}}}$ modes since these decays are dictated by the bottom Yukawa coupling. From $\tan \beta = 10$ to 50, $Br({\mathtt{{B1}} \rightarrow \mathtt{{b N1}}})$ changes from 0 to 28 % (for $M_{Q_3} \simeq 500$ GeV). Because of this effect, the constraint from ATLAS_CONF_2013_053 gets stronger, whilst that from ATLAS_CONF_2013_024 gets weaker as $\tan \beta$ increases. Consequently, the gap is closed for $\tan \beta \gtrsim 40$. In the large $M_{Q_3}$ region, the strongest limit comes from ATLAS_CONF_2013_024 which is designed for ${\mathtt{{T1}} \rightarrow \mathtt{{t N1}}}$ modes. By varying $\tan \beta$ from 10 to 50, the $M_{Q_3}$ limit changes from 750 to 620 GeV.

**Fig. 13:**
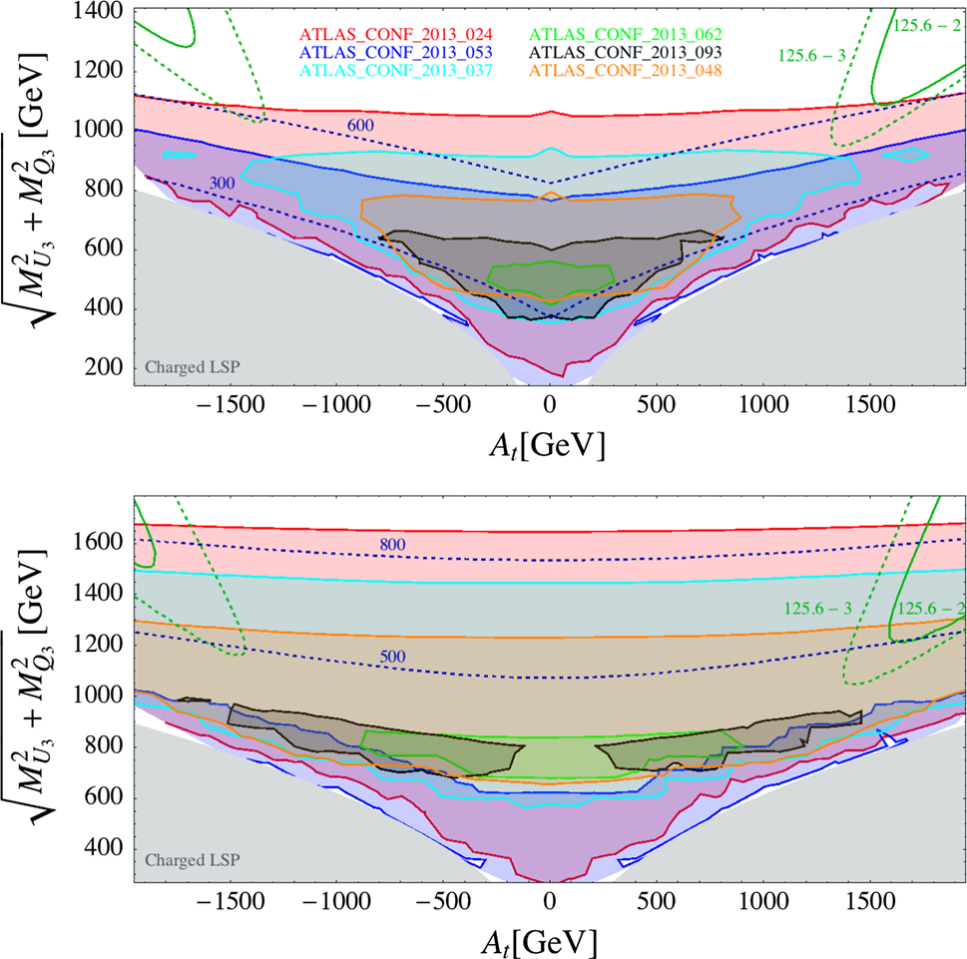
Constraints from direct SUSY searches on the ($A_t$, $(M^2_{U_3} + M^2_{Q_3})^{1/2}$) plane. The *upper plot* we choose $M_{U_3}=M_{Q_3}$ and in the *lower one* $M_{U_3}=2M_{Q_3}$. The other parameters are $m_{\tilde{g}}=M_{D_3}=3{,}000$ GeV, $\tan \beta = 10,\mu =100$ GeV. Both plots show the exclusion regions from the analyses listed in the *upper plot*. The *blue dashed curves* show the ${\tilde{t}}_1$ mass contours. The *green curves* represent the Higgs mass contours, where we allow 3 (*dashed*) and 2 (*solid*) GeV deviation from the central observed value 125.6 GeV

We finally show the exclusion on the ($$A_t$$, $$(M^2_{U_3} + M^2_{Q_3})^{1/2} $$) parameter plane in Fig. 13. In this plane the distance from the origin roughly corresponds to the size of the fine tuning, because the radiative correction to the up-type Higgs soft mass term is given by^11^ [90]

7 $$\begin{aligned} \delta m^2_{H_u} \simeq - \frac{3 y_t^2}{8 \pi ^2} \big ( M_{U_3}^2 + M_{Q_3}^2 + |A_t|^2 \big ) \log \left( \frac{\Lambda }{m_{{\tilde{t}}}}\right) , \end{aligned}$$

where $\Lambda$ is the scale at which the SUSY breaking is mediated in the MSSM sector. We take $M_{U_3} = M_{Q_3}$ in the upper panel, whereas $M_{U_3} = 2 \,M_{Q_3}$ in the lower panel. The other parameters are $\mu = 100$ GeV, $\tan \beta = 10$.

As can be seen, ATLAS_CONF_2013_024 again places the most stringent limit on the soft mass for the third generation squarks for both the $$M_{U_3}/M_{Q_3} = 1$$ and the $$ = 2$$ cases. The blue dashed curves show the $${\tilde{t}}_1$$ mass contours. One can see that the exclusion limit on $$(M^2_{U_3} + M^2_{Q_3})^{1/2}$$ does not change much when $$A_t$$ is varied, although the limit on the $${\tilde{t}}_1$$ mass changes from 780 to 600 GeV as $$|A_t|$$ changes from 0 to 2 TeV (for $$(M^2_{U_3}+M^2_{Q_3})^{1/2} \simeq 1$$ TeV) in the $$M_{U_3}/M_{Q_3} = 1$$ scenario. Increasing $$|A_t|$$ results in making the mass splitting between $${\tilde{t}}_1$$ and $${\tilde{t}}_2$$ larger. However, the changes in the cross section times efficiency from the $${\tilde{t}}_1 {\tilde{t}}_1^*$$ and $${\tilde{t}}_2 {\tilde{t}}_2^*$$ processes tend to cancel each other and the resulting visible cross sections are more or less stable against the variation of $$|A_t|$$. For $$M_{U_3}/M_{Q_3} = 2$$ scenario, $${\tilde{t}}_1$$ is mostly composed of $${\tilde{t}}_L$$ and the dependence of $$|A_t|$$ on the $${\tilde{t}}_1$$ mass itself is very mild.

The green curves represent the Higgs mass contours, where we allow 3 (dashed) and 2 (solid) GeV deviation from the central observed value, taking the theory uncertainties into account. We have calculated the Higgs mass using FeynHiggs 2.9.4 [91]. Most of the parameter space is constrained by the Higgs mass measurement in the $M_{U_3}/M_{Q_3} = 1$ scenario, whereas in the $M_{U_3}/M_{Q_3} = 2$ scenario the ATLAS_CONF_2013_024 analysis excludes (at $95~\%$ CL) a significant part of the parameter space where the Higgs mass condition is satisfied.

## Discussion and future developments

In this paper we presented a program (Fastlim) which calculates the constraints from direct SUSY collider searches starting from a given SLHA model input file. A novel feature of the program is that it does not run any MC simulation to calculate the visible cross section. The program instead *reconstructs* the visible cross section for each signal region by adding the contributions from various event topologies. The cross section and efficiencies for each event topology and each search signal region are obtained by interpolating the pre-calculated cross section and efficiency tables. Similar ideas have also been discussed in the literature [1, 3, 92, 93].

A similar but different approach has recently been taken and implemented in [94]. In this approach, one checks if the model contains the event topologies on which the cross section upper limit is reported by the experimental collaborations.^12^ If such event topologies are found, the program calculates the cross section time branching ratios for those topologies and if one of them exceeds the experimental upper limit, it declares the model to be excluded. This method provides generally weaker but more conservative limits compared to our approach (assuming the same analyses are tested) since there is no attempt made to reconstruct the full BSM contribution to each signal region.

To implement our visible cross section reconstruction method, we have introduced a minimal and intuitive naming scheme for the event topology, which can also be conveniently used as a directory or file name for the efficiency tables. We have also introduced useful approximations which are used to enhance the applicability and speed of the program. Such approximations include shortening the decay chains in presence of mass degeneracies in the spectrum, or recycling efficiency maps in presence of different SUSY particles sharing similar decay modes.

To demonstrate the utility of the program, we have studied the direct SUSY search constraints on natural SUSY models. Using the results of the 2013 ATLAS SUSY searches, we have found that the stop is excluded up to about $700$ GeV with $\mu \lesssim 200$ GeV, whereas the gluino mass is excluded up to about $1.2$ TeV with $\mu \lesssim 400$ GeV. When $A_t$ is varied, we found that the direct SUSY search constraint can be more stringent compared to the Higgs mass constraint in some parameter region, which was not the case when the 7 TeV data was considered [56]. Running Fastlim to extract the limits on the 4,836 parameter points composing the two-dimensional plots shown in this paper took 18.7 h (14 s per point on average) on a single computer (single core, 2.4 GHz clock speed).

Fastlim version 1.0 contains the set of event topologies shown in Fig. 7. These topologies cover the natural SUSY model parameter space very well but they can also cover other models such as split SUSY models with a decoupled wino or bino. More topologies and analyses will be implemented in future updates very soon, thus extending the range of applicability of the approach. The code structure is flexible and the efficiency tables provided from other collaborations can be included straightforwardly (the steps necessary to include a new efficiency table are given in Appendix ). We particularly hope that the experimental collaborations will directly provide their efficiencies in a table format so that the results can be included and thus reinterpreted in a wide range of the SUSY models. Recasting LHC analyses to extend the number of topologies covered is becoming a coordinated effort [80]. Once enough topologies will be available Fastlim can be used for computationally lean pMSSM studies, which may give new insights into interesting SUSY models based on the LHC data.

## Figures and Tables

**Fig. 14:**
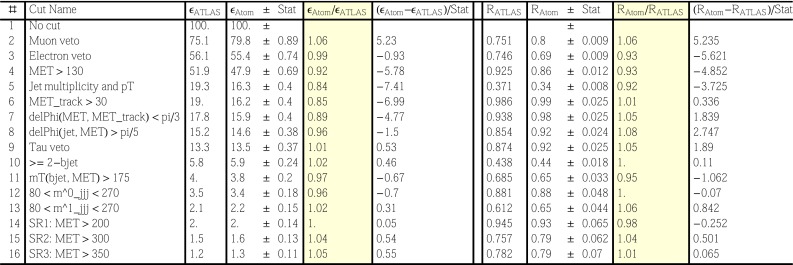
The efficiencies in the cut-flow for ATLAS_CONF_2013_024. $10^4$ events of $pp \rightarrow {\tilde{t}}_R {\tilde{t}}_R^* \rightarrow t \tilde{\chi }_1^0 \bar{t} \tilde{\chi }_1^0$ process are used. The stop and neutralino masses are 600 and 0 GeV, respectively

**Fig. 15:**
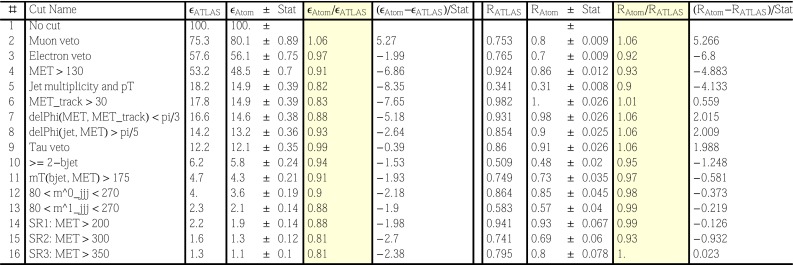
The same as in Fig. 14 but with ${\tilde{t}}_L {\tilde{t}}_L^*$

**Fig. 16:**
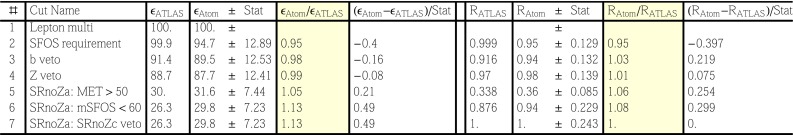
“noZa” signal region in ATLAS_CONF_2013_035. $10^3$ events of $pp \rightarrow {\tilde{\chi }_{1}^{\pm }}{\tilde{\chi }_{2}^{0}}$ process, followed by ${\tilde{\chi }_{1}^{\pm }}\rightarrow \ell ^\pm \nu {\tilde{\chi }_{1}^{0}}$ and ${\tilde{\chi }_{2}^{0}}\rightarrow \ell ^+ \ell ^- {\tilde{\chi }_{1}^{0}}$ both via an on-shell ${\tilde{\ell }}_L$, are used. The masses are $m_{\tilde{\chi }_{1}^{\pm }}= m_{\tilde{\chi }_{2}^{0}}= 192.5$ GeV, $m_{{\tilde{\ell }}_L} = 175$ GeV, $m_{\tilde{\chi }_{1}^{0}}= 157.5$ GeV

**Fig. 17:**
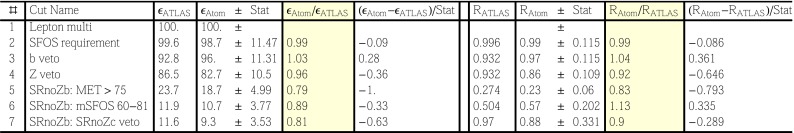
“noZb” signal region in ATLAS_CONF_2013_035. $10^4$ events of $pp \rightarrow {\tilde{\chi }_{1}^{\pm }}{\tilde{\chi }_{2}^{0}}\rightarrow W^\pm {\tilde{\chi }_{1}^{0}}Z {\tilde{\chi }_{1}^{0}}$ process are used. The masses are: $m_{\tilde{\chi }_{1}^{\pm }}= m_{\tilde{\chi }_{2}^{0}}= 150$ GeV, $m_{\tilde{\chi }_{1}^{0}}= 75$ GeV

**Fig. 18:**
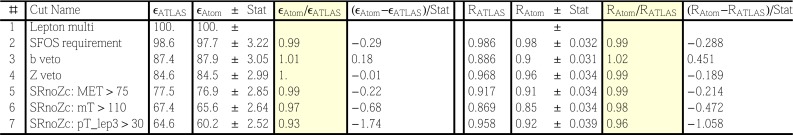
“noZc” signal region in ATLAS_CONF_2013_035. $5 \times 10^3$ events of $pp \rightarrow {\tilde{\chi }_{1}^{\pm }}{\tilde{\chi }_{2}^{0}}$ process, followed by ${\tilde{\chi }_{1}^{\pm }}\rightarrow \ell ^\pm \nu {\tilde{\chi }_{1}^{0}}$ and ${\tilde{\chi }_{2}^{0}}\rightarrow \ell ^+ \ell ^- {\tilde{\chi }_{1}^{0}}$ both via an on-shell ${\tilde{\ell }}_L$, are used. The masses are: $m_{\tilde{\chi }_{1}^{\pm }}= m_{\tilde{\chi }_{2}^{0}}= 500$ GeV, $m_{{\tilde{\ell }}_L} = 250$, $m_{\tilde{\chi }_{1}^{0}}= 0$ GeV

**Fig. 19:**
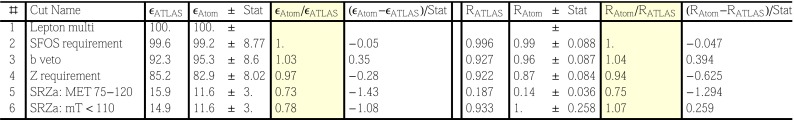
“Za” signal region in ATLAS_CONF_2013_035. $2 \times 10^4$ events of $pp \rightarrow {\tilde{\chi }_{1}^{\pm }}{\tilde{\chi }_{2}^{0}}\rightarrow W^\pm {\tilde{\chi }_{1}^{0}}Z {\tilde{\chi }_{1}^{0}}$ process are used. The masses are: $m_{\tilde{\chi }_{1}^{\pm }}= m_{\tilde{\chi }_{2}^{0}}= 100$ GeV, $m_{\tilde{\chi }_{1}^{0}}= 0$ GeV

**Fig. 20:**
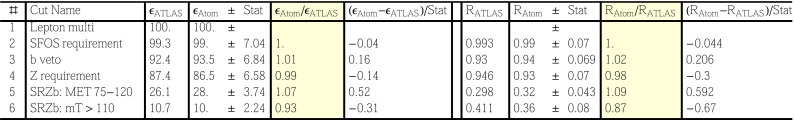
“Zb” signal region in ATLAS_CONF_2013_035. $3 \times 10^4$ events of $pp \rightarrow {\tilde{\chi }_{1}^{\pm }}{\tilde{\chi }_{2}^{0}}\rightarrow W^\pm {\tilde{\chi }_{1}^{0}}Z {\tilde{\chi }_{1}^{0}}$ process are used. The masses are: $m_{\tilde{\chi }_{1}^{\pm }}= m_{\tilde{\chi }_{2}^{0}}= 150$ GeV, $m_{\tilde{\chi }_{1}^{0}}= 0$ GeV

**Fig. 21:**
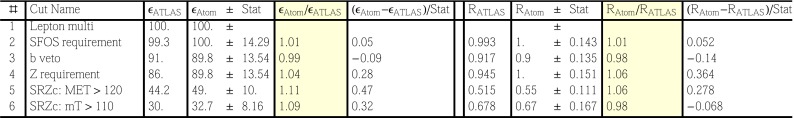
“Zc” signal region in ATLAS_CONF_2013_035. $5 \times 10^3$ events of $pp \rightarrow {\tilde{\chi }_{1}^{\pm }}{\tilde{\chi }_{2}^{0}}\rightarrow W^\pm {\tilde{\chi }_{1}^{0}}Z {\tilde{\chi }_{1}^{0}}$ process are used. The masses are: $m_{\tilde{\chi }_{1}^{\pm }}= m_{\tilde{\chi }_{2}^{0}}= 250$ GeV, $m_{\tilde{\chi }_{1}^{0}}= 0$ GeV

**Fig. 22:**
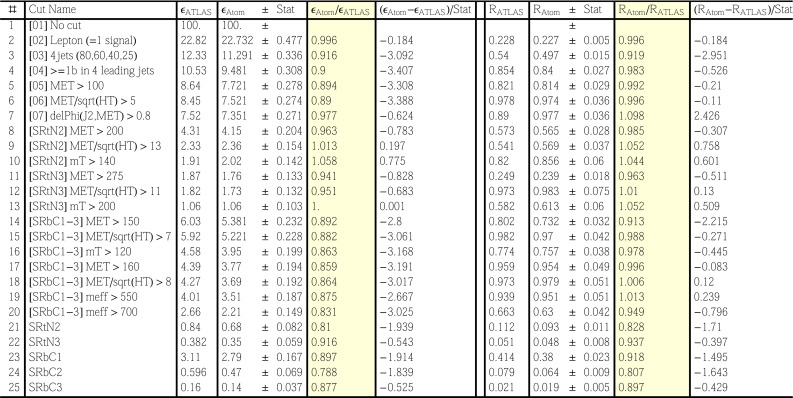
ATLAS_CONF_2013_037 validation table. $10^4$ events of $pp \rightarrow {\tilde{t}}_1 {\tilde{t}}_1^* \rightarrow t {\tilde{\chi }_{1}^{0}}\bar{t} {\tilde{\chi }_{1}^{0}}$ process are used with $m_{{\tilde{t}}_1} = 500$ GeV and $m_{\tilde{\chi }_{1}^{0}}= 200$ GeV. In the cut stages 8, 14 and 21–25, $R_\mathrm{ATLAS/ATOM}$ is defined as the efficiency normalised by the efficiency at the stage 7

**Fig. 23:**
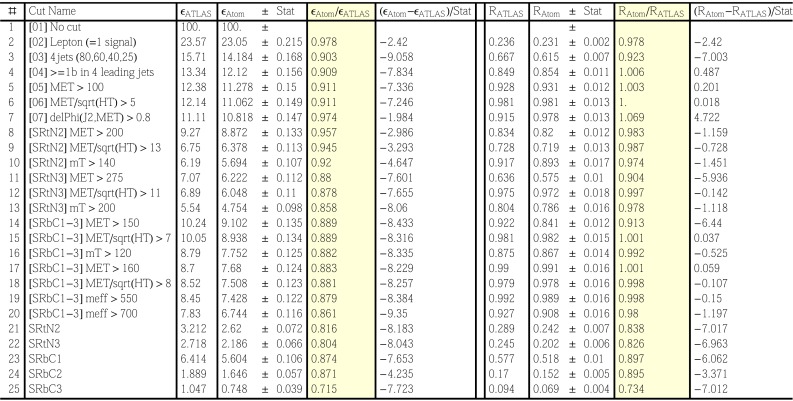
The same as Fig. 22 but $5 \times 10^4$ events of $pp \rightarrow {\tilde{t}}_1 {\tilde{t}}_1^* \rightarrow t {\tilde{\chi }_{1}^{0}}{\bar{t}} {\tilde{\chi }_{1}^{0}}$ process with $m_{{\tilde{t}}_1} = 650$ GeV and $m_{\tilde{\chi }_{1}^{0}}= 1$ GeV

**Fig. 24:**
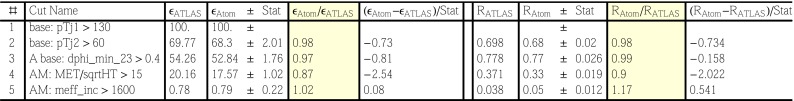
“A medium” signal region in ATLAS_CONF_2013_047. $2 \times 10^4$ events of $pp \rightarrow {\tilde{q}} {\tilde{q}} \rightarrow q {\tilde{\chi }_{1}^{0}}q {\tilde{\chi }_{1}^{0}}$ process are used. The masses are: $m_{{\tilde{q}}} = 450$ GeV, $m_{\tilde{\chi }_{1}^{0}}= 400$ GeV

**Fig. 25:**
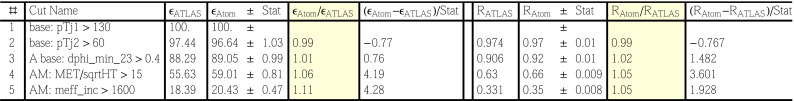
“A medium” signal region in ATLAS_CONF_2013_047. $10^4$ events of $pp \rightarrow {\tilde{q}} {\tilde{q}} \rightarrow q {\tilde{\chi }_{1}^{0}}q {\tilde{\chi }_{1}^{0}}$ process are used. The masses are: $m_{{\tilde{q}}} = 850$ GeV, $m_{\tilde{\chi }_{1}^{0}}= 100$ GeV

**Fig. 26:**
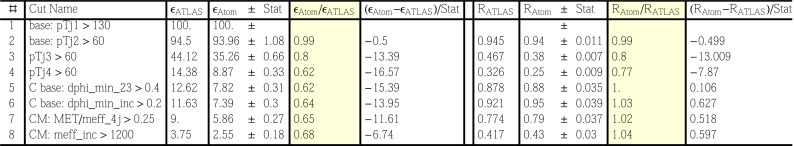
“C medium” signal region in ATLAS_CONF_2013_047. $10^4$ events of $pp \rightarrow {\tilde{q}} {\tilde{q}} \rightarrow q {\tilde{\chi }_{1}^{0}}q {\tilde{\chi }_{1}^{0}}$ process are used. The masses are: $m_{{\tilde{q}}} = 662$ GeV, $m_{\tilde{\chi }_{1}^{0}}= 287$ GeV

**Fig. 27:**
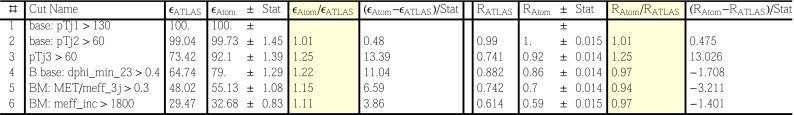
“B medium” signal region in ATLAS_CONF_2013_047. $5 \times 10^3$ events of $pp \rightarrow {\tilde{g}} {\tilde{q}}$ process, followed by ${\tilde{g}} \rightarrow q \bar{q} {\tilde{\chi }_{1}^{0}}$ and ${\tilde{q}} \rightarrow q {\tilde{\chi }_{1}^{0}}$, are used. The masses are: $m_{\tilde{g}}= 1{,}425$ GeV, $m_{{\tilde{q}}} = 1{,}368$ GeV and $m_{\tilde{\chi }_{1}^{0}}= 525$ GeV

**Fig. 28:**
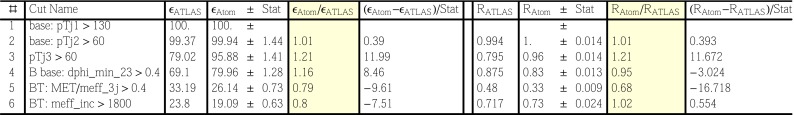
“B tight” signal region in ATLAS_CONF_2013_047. $5 \times 10^3$ events of $pp \rightarrow {\tilde{g}} {\tilde{q}}$ process, followed by ${\tilde{g}} \rightarrow q \bar{q} {\tilde{\chi }_{1}^{0}}$ and ${\tilde{q}} \rightarrow q {\tilde{\chi }_{1}^{0}}$, are used. The masses are: $m_{\tilde{g}}= 1{,}612$ GeV, $m_{{\tilde{q}}} = 1{,}548$ GeV and $m_{\tilde{\chi }_{1}^{0}}= 37$ GeV

**Fig. 29:**
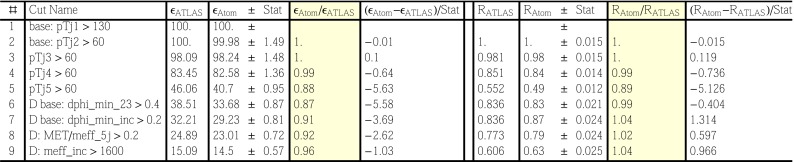
“D” signal region in ATLAS_CONF_2013_047. $5 \times 10^3$ events of $pp \rightarrow {\tilde{g}} {\tilde{g}} \rightarrow qq {\tilde{\chi }_{1}^{0}}qq {\tilde{\chi }_{1}^{0}}$ process are used. The masses are: $m_{\tilde{g}}= 1{,}162$ GeV and $m_{\tilde{\chi }_{1}^{0}}= 337$ GeV

**Fig. 30:**
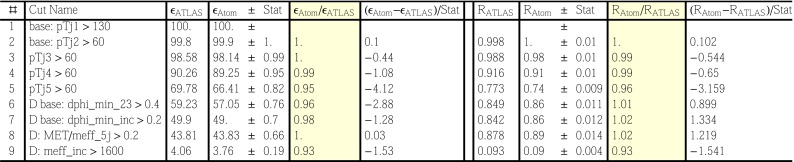
“D” signal region in ATLAS_CONF_2013_047. $2 \times 10^4$ events of $pp \rightarrow {\tilde{g}} {\tilde{g}}$ followed by ${\tilde{g}}\rightarrow qq {\tilde{\chi }_{1}^{\pm }}\rightarrow qq W^\pm {\tilde{\chi }_{1}^{0}}$ are used. The masses are: $m_{\tilde{g}}= 1{,}065$ GeV, $m_{\tilde{\chi }_{1}^{\pm }}= 785$ GeV and $m_{\tilde{\chi }_{1}^{0}}= 505$ GeV

**Fig. 31:**
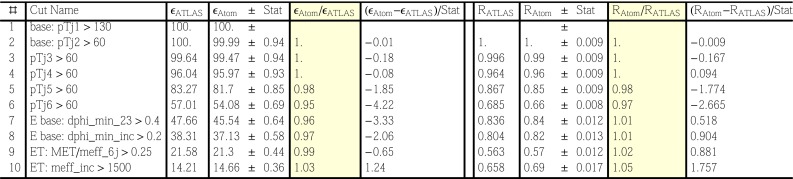
“E tight” signal region in ATLAS_CONF_2013_047. $2 \times 10^4$ events of $pp \rightarrow {\tilde{g}} {\tilde{g}}$ followed by ${\tilde{g}}\rightarrow qq {\tilde{\chi }_{1}^{\pm }}\rightarrow qq W^\pm {\tilde{\chi }_{1}^{0}}$ are used. The masses are: $m_{\tilde{g}}= 1{,}265$ GeV, $m_{\tilde{\chi }_{1}^{\pm }}= 865$ GeV and $m_{\tilde{\chi }_{1}^{0}}= 465$ GeV

**Fig. 32:**
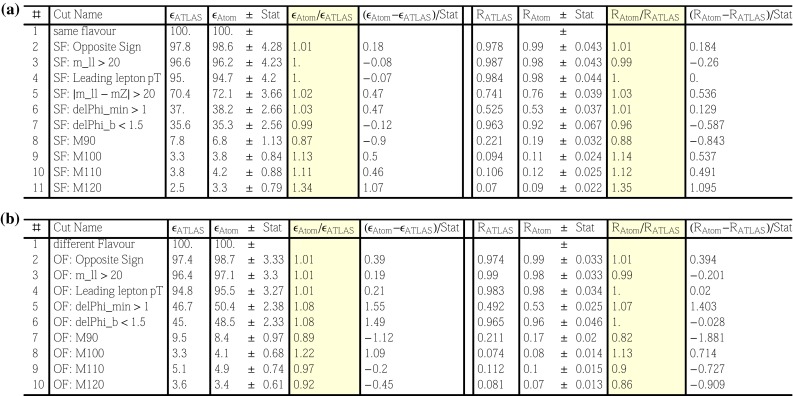
The signal regions in the same (**a**) and opposite (**b**) flavour channels in ATLAS_CONF_2013_048. $3\times 10^4$ events of $pp \rightarrow {\tilde{t}}_1 {\tilde{t}}_1^*$ followed by ${\tilde{t}}_1 \rightarrow b {\tilde{\chi }_{1}^{\pm }}\rightarrow b W^+ {\tilde{\chi }_{1}^{0}}$ are used. The masses are: $m_{{\tilde{t}}_1} = 400$ GeV, $m_{\tilde{\chi }_{1}^{\pm }}= 250$ GeV and $m_{\tilde{\chi }_{1}^{0}}= 1$ GeV

**Fig. 33:**
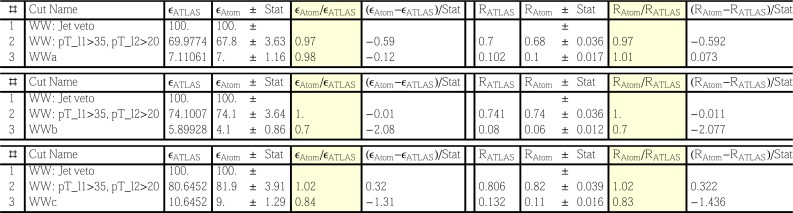
“Wa” (*top*), “Wb” (*middle*) and “Wc” (*bottom*) signal regions in ATLAS_CONF_2013_049. $5\times 10^4$ events of $pp \rightarrow {\tilde{\chi }_{1}^{+}}{\tilde{\chi }_{1}^{-}}\rightarrow W^+ {\tilde{\chi }_{1}^{0}}W^- {\tilde{\chi }_{1}^{0}}$ process are used. The masses are: $(m_{\tilde{\chi }_{1}^{\pm }}, m_{\tilde{\chi }_{1}^{0}}) = (100, 0)$ GeV for Wa, $(m_{\tilde{\chi }_{1}^{\pm }}, m_{\tilde{\chi }_{1}^{0}}) = (140, 20)$ GeV for Wb and $(m_{\tilde{\chi }_{1}^{\pm }}, m_{\tilde{\chi }_{1}^{0}}) = (200, 0)$ GeV for Wc

**Fig. 34:**
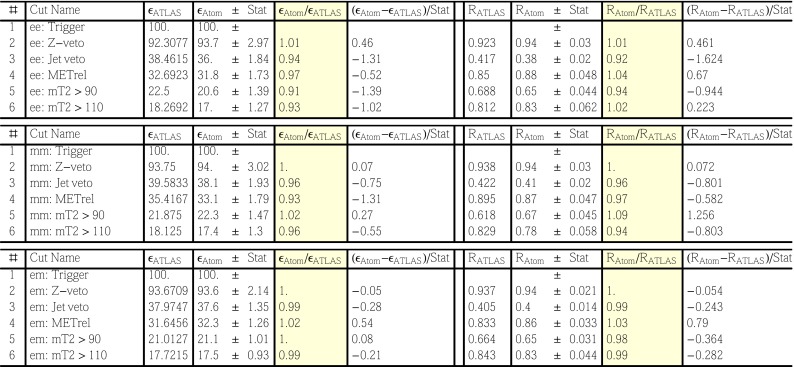
“mT2:90” and “mT2:100” signal regions in the $ee$ (*top*), $\mu \mu$ (*middle*) and $e \mu$ (*bottom*) channels in ATLAS_CONF_2013_049. $10^4$ events of $pp \rightarrow {\tilde{\chi }_{1}^{+}}{\tilde{\chi }_{1}^{-}}$ process, followed by ${\tilde{\chi }_{1}^{\pm }}\rightarrow \ell ^\pm _i \nu {\tilde{\chi }_{1}^{0}}$ via an on-shell ${\tilde{\ell }}_i$, are used. The masses are: $m_{\tilde{\chi }_{1}^{\pm }}= 350$ GeV, $m_{{\tilde{\ell }}} = 175$ GeV and $m_{\tilde{\chi }_{1}^{0}}= 0$ GeV

**Fig. 35:**
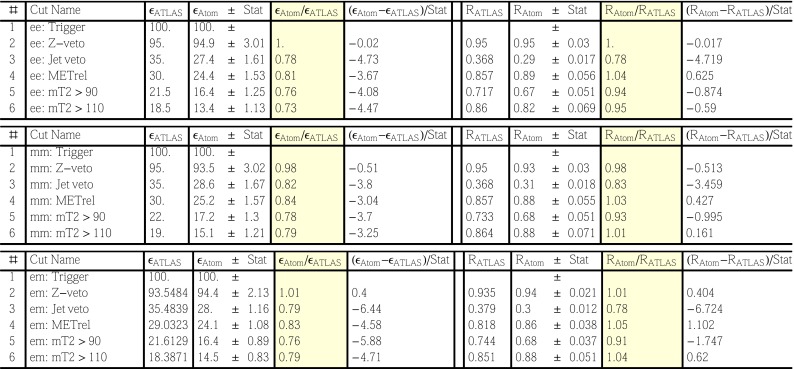
The same as Fig. 34 but $m_{\tilde{\chi }_{1}^{\pm }}= 425$ GeV, $m_{{\tilde{\ell }}} = 250$ GeV and $m_{\tilde{\chi }_{1}^{0}}= 75$ GeV

**Fig. 36:**
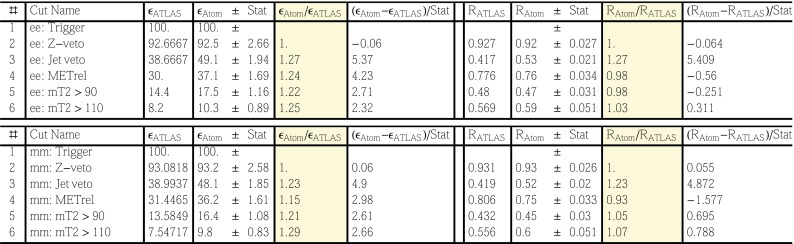
“mT2:90” and “mT2:100” signal regions in the $ee$ (*top*) and $\mu \mu$ (*middle*) channels in ATLAS_CONF_2013_049. $2\times 10^3$ events of $pp \rightarrow {\tilde{e}}^+ {\tilde{e}}^- \rightarrow e^+ {\tilde{\chi }_{1}^{0}}e^- {\tilde{\chi }_{1}^{0}}$ and $pp \rightarrow {\tilde{\mu }}^+ {\tilde{\mu }}^- \rightarrow \mu ^+ {\tilde{\chi }_{1}^{0}}\mu ^- {\tilde{\chi }_{1}^{0}}$ processes are used for $ee$ and $\mu \mu$ channels, respectively. The masses are: $m_{{\tilde{\ell }}} = 191$ GeV and $m_{\tilde{\chi }_{1}^{0}}= 90$ GeV

**Fig. 37:**
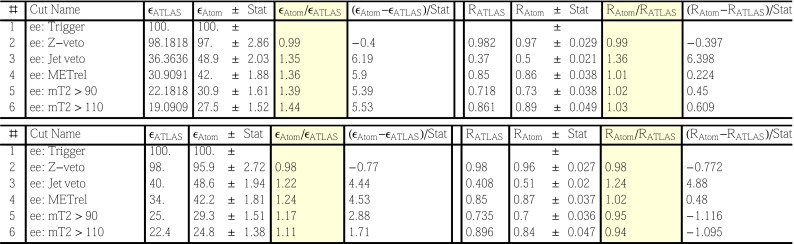
The same as Fig. 37 but $m_{{\tilde{\ell }}} = 250$ GeV and $m_{\tilde{\chi }_{1}^{0}}= 10$ GeV

**Fig. 38:**
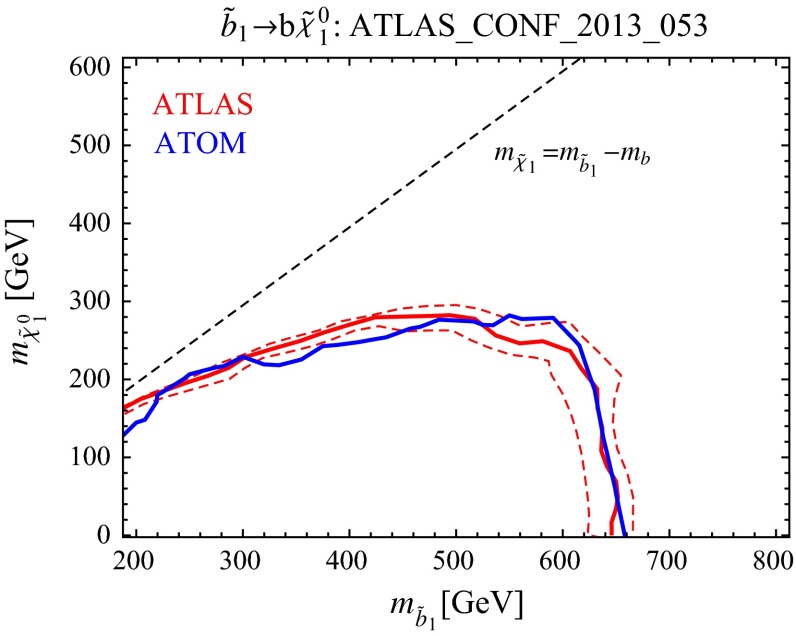
The exclusion curves in the ${\tilde{b}}_1$–${\tilde{\chi }_{1}^{0}}$ simplified model parameter space (${\tilde{b}}_1 \rightarrow b {\tilde{\chi }_{1}^{0}}$). The *red* and *blue curves* are for ATLAS and ATOM, respectively. The *red dashed curves* show the 1-$\sigma$ error band of the ATLAS exclusion curve

**Fig. 39:**
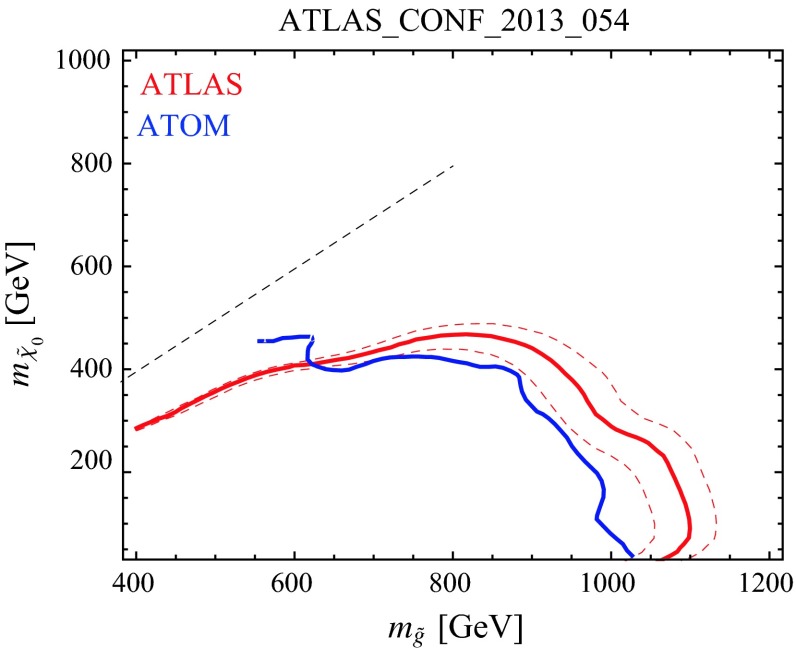
The exclusion curves in the ${\tilde{g}}$–${\tilde{\chi }_{1}^{\pm }}$–${\tilde{\chi }_{1}^{0}}$ simplified model parameter space (${\tilde{g}} \rightarrow qq {\tilde{\chi }_{1}^{\pm }}\rightarrow qq W^\pm {\tilde{\chi }_{1}^{0}}$). The chargino mass fixed at $(m_{\tilde{g}}- m_{\tilde{\chi }_{1}^{0}})/2$. The *red* and *blue curves* are for ATLAS and ATOM, respectively. The *red dashed curves* show the 1-$\sigma$ error band of the ATLAS exclusion curve

**Fig. 40:**
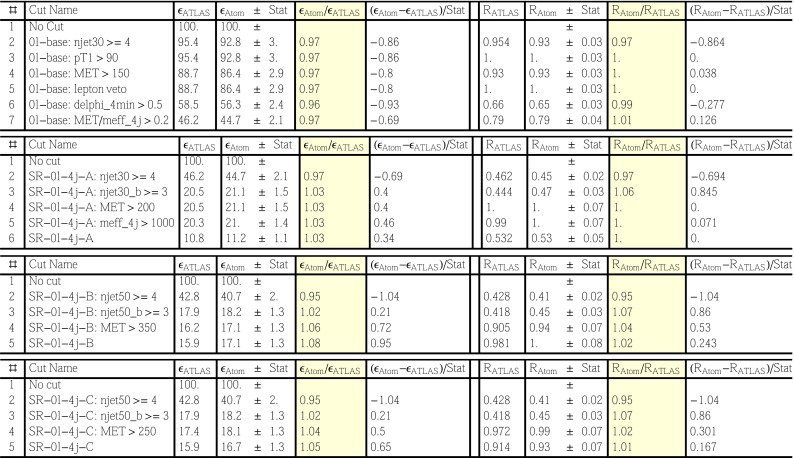
The 0-lepton $+$ 4-jet signal regions in ATLAS_CONF_2013_061. $10^3$ events of $pp \rightarrow {\tilde{g}}{\tilde{g}}\rightarrow b {\bar{b}} {\tilde{\chi }_{1}^{0}}b {\bar{b}} {\tilde{\chi }_{1}^{0}}$ process generated by MadGraph 5 are used. The masses are: $m_{\tilde{g}}= 1{,}300$ GeV and $m_{\tilde{\chi }_{1}^{0}}= 100$ GeV

**Fig. 41:**
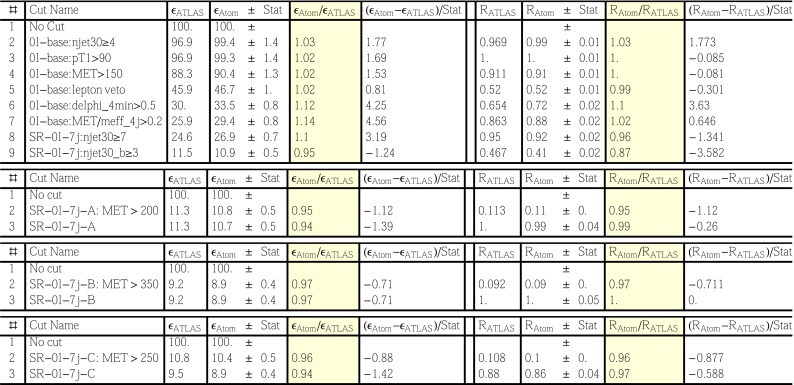
The 0-lepton + 7-jet signal regions in ATLAS_CONF_2013_061. $5\times 10^3$ events of $pp \rightarrow {\tilde{g}}{\tilde{g}}\rightarrow t \bar{t} {\tilde{\chi }_{1}^{0}}t \bar{t} {\tilde{\chi }_{1}^{0}}$ process generated by Herwig++ 2.5.2 are used. The masses are: $m_{\tilde{g}}= 1{,}300$ GeV and $m_{\tilde{\chi }_{1}^{0}}= 100$ GeV

**Fig. 42:**
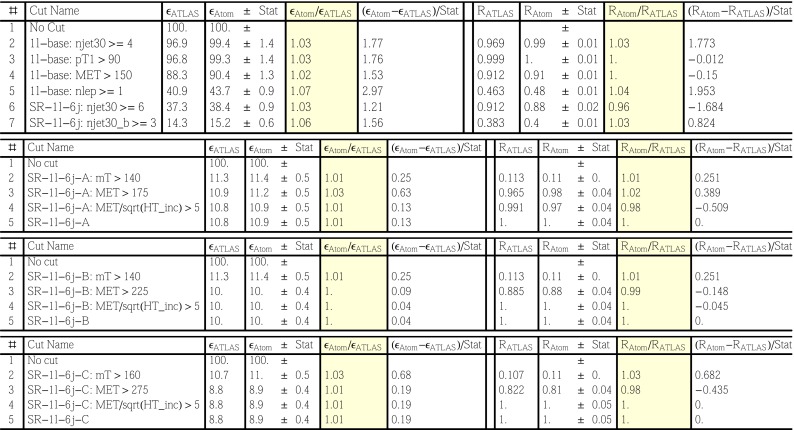
The 1-lepton $$ +$$ 6-jet signal regions in ATLAS_CONF_2013_061. $$5\times 10^3$$ events of $$pp \rightarrow {\tilde{g}}{\tilde{g}}\rightarrow t \bar{t} {\tilde{\chi }_{1}^{0}}t \bar{t} {\tilde{\chi }_{1}^{0}}$$ process generated by Herwig++ 2.5.2 are used. The masses are: $$m_{\tilde{g}}= 1{,}300$$ GeV and $$m_{\tilde{\chi }_{1}^{0}}= 100$$ GeV

**Fig. 43:**
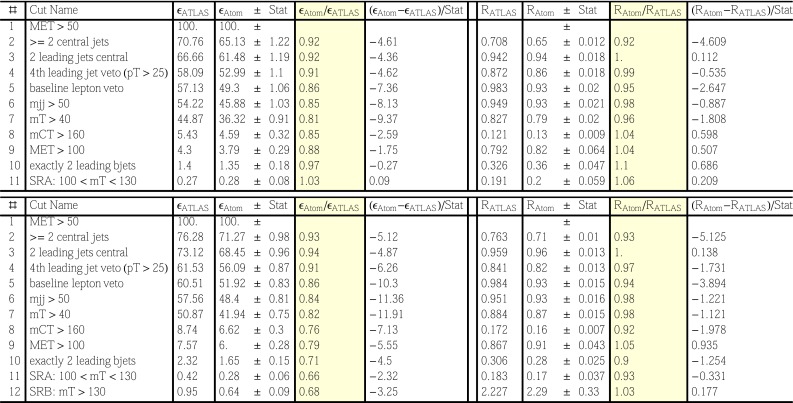
The signal regions in ATLAS_CONF_2013_093. $5 \times 10^4$ events of $pp \rightarrow {\tilde{\chi }_{1}^{\pm }}{\tilde{\chi }_{2}^{0}}\rightarrow W^\pm {\tilde{\chi }_{1}^{0}}h^0 {\tilde{\chi }_{1}^{0}}$ process are used. The masses are: $m_{\tilde{\chi }_{1}^{\pm }}= m_{\tilde{\chi }_{2}^{0}}= 130 (225)$ GeV, $m_{\tilde{\chi }_{1}^{0}}= 0$ GeV for the *top* (*bottom*) table

## Appendix A: Installation

To run Fastlim on your system, first download the latest version of the program via:

http://cern.ch/fastlim

Fastlim is based on the following software:

- Python [95], typically preinstalled;
- NumPy [96] and SciPy [97], whose installation is recommended.

Fastlim was developed using Python version 2.7, NumPy version 1.7.1 and SciPy version 0.12.0.^13^ The default interpolation routine in Fastlim uses NumPy and SciPy. If these packages are not available, Fastlim switches to a cruder nearest-neighbour interpolation. The $CL_s$ calculation relies on NumPy. If the user is only interested in the $R^{(a)}$ values, it is possible to use Fastlim without NumPy/SciPy; however, we strongly recommend to install these packages. Details of the installation of NumPy/SciPy can be found on the Fastlim website. After downloading, run the commands  to extract the tarball and enter the directory. No further installation is required.

Bugs and feature requests may be reported by sending an email to fastlim.developers@gmail.com.

## Appendix B: Quick start

After the installation, the program can be executed by

where testspectrum.slha is a sample SLHA spectrum file, which can be found in the slha_files directory. A short summary of the results will be displayed on the screen and the output file fastlim.out will be created. If users want to run multiple spectrum files placed under slha_files, the preferred way is via the command

In this case, the output files will be created and stored in the ScanOutput directory.

## Appendix C: Implementing efficiency tables provided by other collaborations

Efficiency tables provided in data format by other collaborations or directly by ATLAS or CMS can straightforwardly be included in Fastlim. In order to demonstrate this feature, we describe how to include the efficiency tables (e.g. for the simplified topologies $\mathtt{{GqqN1\_GqqN1}}$ and $\mathtt{{QqN1\_QqN1}}$) of a 7 TeV ATLAS analysis [98] (0 leptons + 2–6 jets + Etmiss) which are available on HepData [99]. To implement a new efficiency table:

- Create a new folder of the respective topology and analysis, e.g.
- Copy the efficiency tables into this folder (one file for each signal region) and, if necessary, bring it into the format shown in Fig. 6.^14^
- Create a new folder, e.g.  with a file SR_info.txt containing the relevant information, such as the $95~\%$ CL upper limit on the visible cross section, $\sigma ^{(a)}_\mathrm{UL}$, which can be extracted from the experimental paper describing the analysis (e.g. Table III of [98]).

No further modifications of the Fastlim code are necessary.

## Appendix D: Validation

The efficiency tables installed in Fastlim version 1.0 are generated by ATOM [55], in which we have implemented various 2013 ATLAS analyses. We have validated our implementation mostly using the cut-flow tables provided by ATLAS. For ATLAS_CONF_2013_062, the truth-level information is used in the ATLAS cut-flow tables, which prevents us from comparing our efficiencies and ATLAS’s. We validated this analysis among the collaborations by cross checking the two independent implementation of the analysis. For ATLAS_CONF_2013_053 and ATLAS_CONF_2013_054 the cut-flow tables are not provided. We therefore validated them using the simplified model exclusion plots given in the manuscripts [75, 76]. The discrepancies between ATLAS and ATOM are within $10$–$20~\%$ for most of the signal regions. For the worst signal region the disagreement is about $30~\%$. Such deviations often come from jet veto cuts, which possess large theoretical uncertainties. Further tuning of the ATOM detector response may improve the situation. Updated grids and validation tables will be provided in future Fastlim versions and on the website (http://cern.ch/fastlim).

In what follows, we present a normalised efficiency for each stage of the cut in the cut-flow tables. ATLAS sometimes calculates the efficiency after the trigger requirement, whereas we do it before that. For such cases, the comparison is only reasonable after the cut to which the trigger requirement is subjected. The efficiency is therefore normalised to the efficiency of such a cut, which appears first in the table. In the tables, we use the following variables:

$$\begin{aligned} \epsilon _\mathrm{ATLAS/ATOM}:&\mathrm{normalised ~efficiency ~by ~ATLAS/ATOM},\\ R_\mathrm{ATLAS/ATOM}:&\mathrm{the~ efficiency ~ratio ~against ~the~efficiency}\\&\mathrm{of~the~cut~one ~before},\\ \mathrm{Stat}:&\mathrm{the~ Monte ~Carlo ~uncertainty ~for ~the }\\&\mathrm{~ATOM ~efficiency~or~efficiency ~ratio}. \end{aligned}$$

### D.1 ATLAS_CONF_2013_024

- The events are generated using Herwig++ 2.5.2 [49] throughout this analysis (Figs. 14, 15).

### D.2 ATLAS_CONF_2013_035

- The events are generated using Herwig++ 2.5.2 throughout this analysis (Figs. 16, 17, 18, 19, 20, 21).

### D.3 ATLAS_CONF_2013_037

- The events are generated using Herwig++ 2.5.2 throughout this analysis (Figs. 22, 23).

### D.4 ATLAS_CONF_2013_047

- The events are generated using MadGraph 5 [52] and Pythia 6 [50] throughout this analysis.
- The MLM merging [68] is used with the shower-$k_T$ scheme implemented in MadGraph 5 and Pythia 6, where we take $\mathrm{xqcut} = \mathrm{qcut} = m_\mathrm{SUSY}/4$ with $m_\mathrm{SUSY}$ being the mass of the heavier SUSY particles in the production (Figs. 24, 25, 26, 27, 28, 29, 30, 31).

### D.5 ATLAS_CONF_2013_048

- The events are generated using MadGraph 5 and Pythia 6 (Fig. 32).

### D.6 ATLAS_CONF_2013_049

- The events are generated using Herwig++ 2.5.2 throughout this analysis (Figs. 33, 34, 35, 36, 37).

### D.7 ATLAS_CONF_2013_053

- The events are generated using MadGraph 5 and Pythia 6 (Fig. 38).

### D.8 ATLAS_CONF_2013_054

- The events are generated using MadGraph 5 and Pythia 6. The MLM merging is used in MadGraph 5 and Pythia 6 with $\mathrm{xqcut} = \mathrm{qcut} = m_{{\tilde{g}}}/4$ (Fig. 39).

### D.9 ATLAS_CONF_2013_061

See Figs. 40, 41 and 42.

### D.10 ATLAS_CONF_2013_093

- The events are generated using Herwig++ 2.5.2 (Fig. 43).

## Footnotes

1 In this paper the term topology refers to the full decay chain and not the observable final state signature. Please see Sect. 3 for the exact definition.

2 The signal regions are the sets of selection cuts defined in the experimental analyses.

3 This approach works with most of the currently available searches which are “cut-and-count”, but may fail with shape-analysis based searches where adding additional contributions may result in signal shapes more difficult to disentangle from the backgrounds.

4 Similar programs have been put forward [58, 59]. A framework based on the calculation of efficiencies by the experimental collaborations has been presented in [60].

5 In certain cases, topologies with more than three SUSY particles may be approximated by two- or three-dimensional topologies, as described in Sect. 6.

6 We use a linear extrapolation for the $\ln x$, where $x$ is the cross section or the efficiency.

7 We do not consider the SUSY particle decays into three or more SUSY particles.

8 A possibility to account for deviations in Higgs branching ratios from the SM values may easily be accounted in future releases.

9 The names of the topologies after the truncation of soft decays are the only exception where “topology” does not mean the full decay chain anymore.

10 Here (and more generally in the discussion of the plots in this section) the exclusion refers to the $95~\%$ CL exclusion given by the analysis that is most sensitive in that region.

11 This leading logarithmic approximation is generically valid for low scale SUSY breaking mediation models, while corresponding resumed expressions for high scale models can be found.

12 To derive the exclusion, the signal topologies are mapped to the topologies constrained in the experimental analyses. This implies that in some cases (in which the analyses target one topology) the exclusion is made from a single event topology, while in other cases (where the analyses constrain a sum of topologies, e.g. a sum over final state lepton flavours) a few topologies are “combined” correspondingly. No recasting of topologies (see Sect. 2.1) which are not covered by the experiments is performed in this approach. The code has a function similar to the replace function in Fastlim to truncate the soft decays. The check if a model point is explored is done after the truncation and combination.
